# Effects of Magnetite Powder on Microwave Heating Properties and Pavement Performance of Asphalt Mixture

**DOI:** 10.3390/ma18214920

**Published:** 2025-10-28

**Authors:** Haoran Zhu, Yajun Zhang, Feng Hu, Mingming Yu, Wenfeng Wang

**Affiliations:** 1 College of Civil and Transportation Engineering, Hohai University, Nanjing 210024, China; hhuzhangyajun@163.com (Y.Z.); hhuhufeng@163.com (F.H.); 2 Jiangsu Expressway Engineering Maintenance Technology Co., Ltd., Nanjing 210049, China; mmyu2025@yeah.net; 3 JSTI Group Co., Ltd., Nanjing 210017, China; wwf235@jsti.com

**Keywords:** magnetite powder, microwave asphalt mixture, microwave absorption and heating performance, pavement performance, COMSOL simulation

## Abstract

Microwave heating is a method with a uniform heating effect and environmental friendliness in in-place hot recycling, but the microwave absorption capacity of traditional asphalt mixtures is still insufficient. As an excellent microwave-absorbing material, magnetite powder has the characteristics of high temperature resistance, corrosion resistance, and good thermodynamic stability. This study selects it as the microwave-absorbing material, prepares AC (Asphalt Concrete) type and SMA (Stone Mastic Asphalt) type microwave asphalt mixtures by adjusting its content, and investigates its influence on the microwave-heating characteristics and pavement performance of the mixtures. Simulations of the microwave-heating process of AC-type mixtures using COMSOL software (COMSOL Multiphysics 6.2) show that magnetite powder achieves optimal performance in terms of heating effect and economic efficiency when its content is 0.5%. Subsequently, laboratory tests are conducted to study the wave absorption and temperature rise performance of AC and SMA microwave asphalt mixtures; combined with economic factors, the optimal contents of magnetite powder for the two types of mixtures are determined to be 0.5% and 1%, respectively, and at the same time, these results are explained based on multiple physical theories. Furthermore, pavement performance is investigated through laboratory tests, including high-temperature rutting tests, low-temperature bending tests, immersed Marshall tests, and freeze–thaw cycle durability tests, and the results indicate that the high-temperature performance, low-temperature performance, and water stability of the microwave asphalt mixtures all meet the specification requirements for pavement performance. Subsequently, after 15 freeze–thaw cycles, the splitting tensile strength retention rate and stiffness modulus of the two types of mixtures show minimal differences from those of ordinary mixtures, and there is no durability degradation caused by the incorporation of magnetite powder. Finally, outdoor environment verification is carried out, and the results show that under complex conditions such as environmental factors, the wave absorption and temperature rise rates of AC and SMA mixtures at optimal contents are 52.2% and 14.6% higher than those of ordinary AC and SMA asphalt mixtures, respectively. In addition, these microwave asphalt mixtures have the advantages of both sustainability and reduced carbon emissions. By combining simulation methods and experimental verification, this study finally prepared two types of microwave asphalt mixtures with excellent performance, not only improving the microwave absorption and heating performance of asphalt mixtures, but also reducing environmental pollution and energy consumption, which conforms to the development of green transportation.

## 1. Introduction

Milling and resurfacing, a traditional technical solution for asphalt pavement maintenance, not only has a long construction period, but also is prone to causing severe resource waste and environmental pollution, and sometimes even leads to internal road structure damage [1,2]. However, in situ thermal recycling technology [3] has become a research focus in the field of road engineering due to its advantages such as 100% recycling of reclaimed asphalt pavement (RAP) materials from old asphalt pavements, fast construction progress, and minimal impact on traffic [4], Researchers have also proposed various construction schemes [5]. Cao et al. [6] conducted a comparative analysis of the ecological benefits of these two asphalt pavement repair solutions and concluded that in situ thermal recycling technology can reduce environmental impact and cost consumption to a certain extent.

At present, three heating methods are generally used in in situ thermal recycling technology: infrared radiation heating [7], hot air circulation [8], and microwave heating [9]. However, infrared radiation heating and hot air circulation have drawbacks, such as limited heating depth, poor uniformity, and a tendency to cause asphalt aging [10]. In contrast, microwave heating, which heats from the interior of the road, features excellent heating uniformity and high energy efficiency. It can enhance pavement sustainability [11] and represents a newly developed pavement-heating method in research, both domestically and internationally.

The microwave absorption and heating capacity of asphalt mixtures under microwave heating depends on the aggregates used. Noojilla et al. [12] investigated the influence of aggregates on the overall heating characteristics of asphalt mixtures and developed two predictive models to estimate the heating rate of aggregates. However, under normal circumstances, the microwave absorption and heating performance of aggregates contained in asphalt mixtures are relatively poor [13], which, to some extent, limits the application effect of microwave heating in the field of in situ thermal recycling. To tackle this problem, researchers have incorporated various microwave-absorbing materials into asphalt mixtures to enhance their microwave absorption and heating capacity under microwave heating. Liu et al. [14] used methods such as X-ray Diffraction, microwave heating cycles, and Computed Tomography scans to study asphalt mixtures mixed with steel slag, and they found that the addition of steel slag could enhance the microwave absorption and heating capacity of asphalt mixtures to a certain extent. After 40 s of microwave heating, the average temperature of the crack propagation area in the group with 100% steel slag replacement reached 119.5 °C, and this temperature was 201.77% higher than that in the pure limestone group. However, there are two limitations in the application of steel slag. First, the synergy between the porous structure of steel slag itself and local microwave overheating will lead to pore expansion. After 10 microwave cycles, the proportion of large pores with an internal volume larger than 1 mm^3^ in the mixture increases from 0.58% to 1.13%, which further damages the internal structural stability of the mixture and thus reduces the pavement performance and durability of asphalt pavements in practical applications [15]. Li et al. [16] added iron tailing fines to asphalt mixtures to replace limestone filler and comprehensively evaluated the microwave-heating performance, high-temperature stability, and water stability of the asphalt mixtures. They found that its microwave-heating capacity and high-temperature stability increase; after 120 s of microwave heating, the temperature of iron tailing fines reaches 108 °C, and this temperature is 2.1 times that of limestone filler (51.3 °C). However, iron tailing fines have an obvious content threshold limitation. In terms of pavement performance, 20% to 60% of iron tailing fines can increase the high-temperature dynamic stability of the mixture by 4% to 27%. But when the content exceeds 40%, the low-temperature bending strain begins to decrease; the water stability of the mixture with 80% iron tailing fines attenuates significantly, and the Freeze–Thaw Splitting Tensile Strength Ratio is close to the lower limit of the specification, so the pavement performance fails to meet the requirements.

The original aggregates in asphalt mixtures typically serve to densify the structure and stabilize the cementitious system [17]. Replacing these original aggregates with materials such as steel slag and iron tailing sand may lead to a decline in the overall pavement performance of asphalt mixtures [18]. Therefore, some researchers have chosen conductive materials like steel fibers [19] and carbon nanotubes to replace mineral powder, aiming to enhance the microwave heating effect of asphalt mixtures while minimizing impacts on other properties of the mixtures. However, steel fibers tend to agglomerate, leading to excessively high local temperatures. The temperature difference (between these areas) can reach more than 30 °C, and this may even cause accelerated aging of asphalt materials, exerting a certain negative impact [20]. In terms of cost, steel fibers are industrial-grade conductive additives; their market price is approximately 4 to 5 times that of magnetite powder. Additionally, additional stirring and dispersion processes are required, which further increases the engineering cost. However, other conductive materials, such as carbon nanotubes [21], have a good heating effect, but their price is high. The market price of industrial-grade carbon nanotubes in the local area is approximately 800 to 1000 yuan per kilogram, which is 200 to 250 times that of magnetite powder. Moreover, their dispersion process requires specialized equipment, which is not suitable for large-scale road thermal recycling projects.

From a comprehensive perspective, the existing research on microwave-absorbing materials has common bottlenecks: industrial waste residue-based materials, such as steel slag and iron tailing sand, struggle to balance microwave absorption performance and pavement performance; highly conductive synthetic materials, such as steel fibers and carbon nanotubes, are either limited by process compatibility or difficult to scale up due to excessively high costs; while research on natural mineral-based microwave-absorbing materials with stable performance and suitable cost is relatively insufficient, and systematic performance verification and application analysis have not yet been formed. This results in the lack of support from ideal modified materials for the engineering application of microwave asphalt mixtures. To address this, this study focused on the problems of improving the microwave-heating efficiency of asphalt mixtures while maintaining their pavement performance and selected magnetite powder—characterized by stable performance and cost-effectiveness for large-scale engineering applications—as the microwave-absorbing material to be incorporated. First, the heating process of AC-type microwave-modified asphalt mixtures was simulated using COMSOL software to reveal the microwave-heating characteristics of microwave-modified asphalt mixtures with different magnetite powder dosages. Subsequently, practical experiments were conducted to investigate the effect of different magnetite powder dosages on the heating performance of both AC-type and SMA-type microwave-modified asphalt mixtures. Meanwhile, the experimental results were mutually verified with the simulation findings, leading to the determination of the optimal magnetite powder dosages for the two types of mixtures. Additionally, the pavement performance of the asphalt mixtures with the added microwave-absorbing material was evaluated. This study not only provides a high-performance and cost-effective microwave-modified asphalt mixture for pavement thermal recycling projects but also reduces environmental pollution and resource waste.

## 2. Materials and Methods

### 2.1. Raw Materials

#### 2.1.1. Microwave-Absorbing Materials

**Figure 1:**
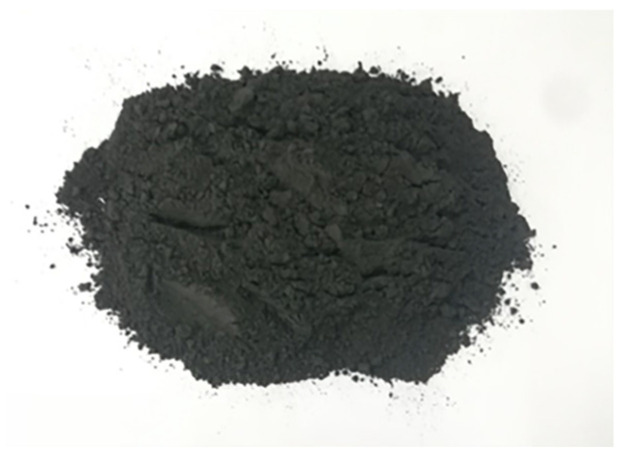
Magnetite powder.

**Figure 2:**
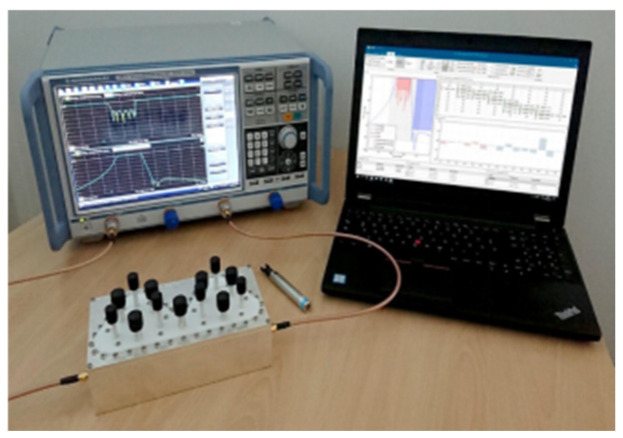
Vector network analyzer.

The magnetite powder used in this study is sourced from Shandong Province, China, and appears as a black powder, as shown in Figure 1. The data of the basic physical indicators of natural magnetite powder obtained through actual measurement are presented in Table 1. Among these parameters, the content of Fe_3_O_4_ is calculated and derived from the contents of total iron (Fe_2_O_3_) and ferrous oxide (FeO). The reference standards are Iron Ores–Determination of Total Iron Content–Titration Method After Titanium Trichloride Reduction (GB/T 6730.5-2016 [22]) and Iron Ores–Determination of Ferrous Oxide Content–Potassium Dichromate Titration Method (GB/T 6730.8-2016 [23]). The complex permittivity is measured using the SVA1000X Series Spectrum & Vector Network Analyzer, the manufacturer of this device is Siglent Technologies Co., Ltd., based in Shenzhen City, China. This analyzer is shown in Figure 2.

To enhance the dispersion of magnetite powder in asphalt mixtures and the adhesion of aggregates, a composite approach involving the incorporation of dispersant, binder, and magnetite powder was adopted. The types and properties of the dispersant and binder are presented in Table 2.

**Figure 3:**
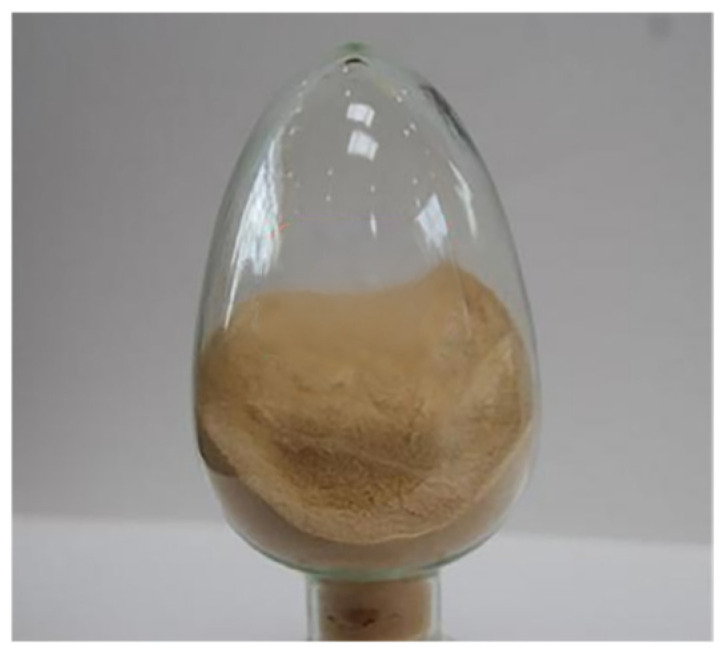
Dispersant.

**Figure 4:**
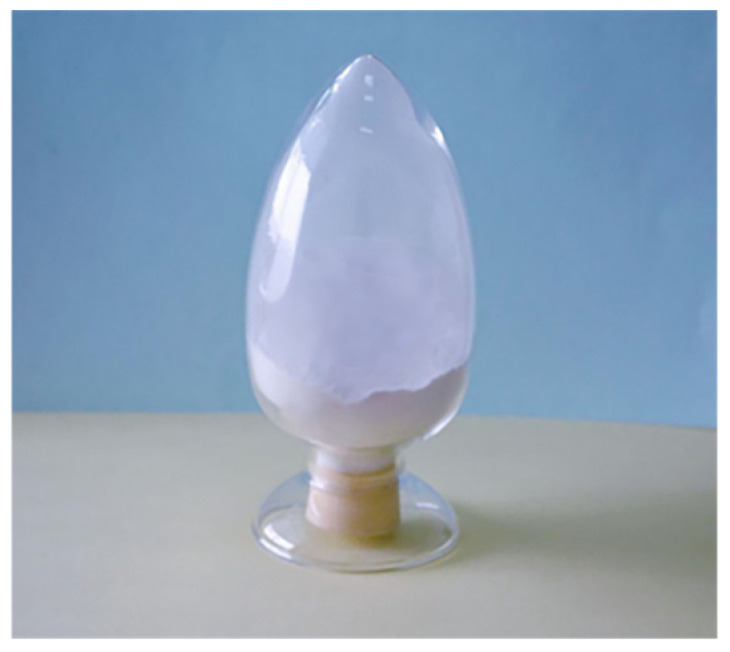
Binder.

The dispersant with a dosage of 0.3% by mass of the microwave-absorbing material and the binder with a dosage of 0.2% by mass of the same material were first mixed with the microwave-absorbing material, after which stirring was conducted using a small-scale remixing machine (The manufacturer of this equipment is Changzhou Guohua Electric Appliance Co., Ltd., located in Changzhou, China)at a rotational speed of 200–300 r/min. Once the three components were uniformly mixed, the mixture was sealed and stored in a cool, shaded indoor area for subsequent use; moisture absorption by the microwave-absorbing material is strictly prohibited. The dispersant and the binder are shown in Figure 3 and Figure 4.

#### 2.1.2. Asphalt Materials

This study uses SBS-modified asphalt of Type I-C produced in Jiangyin Taifu Asphalt Co., Ltd., located in Jiangyin City, China, and the specific test results and technical indicators of this asphalt are presented in Table 3.

#### 2.1.3. Aggregates and Mineral Powders

The coarse aggregates, fine aggregates, and mineral powder used in this study are produced in Hebei Province, China, and the technical indicators of these materials are presented in Table 4, Table 5 and Table 6, respectively.

#### 2.1.4. Preparation of Asphalt Mixture

(1) Preparation of AC-13 Asphalt Mixture

**Figure 5:**
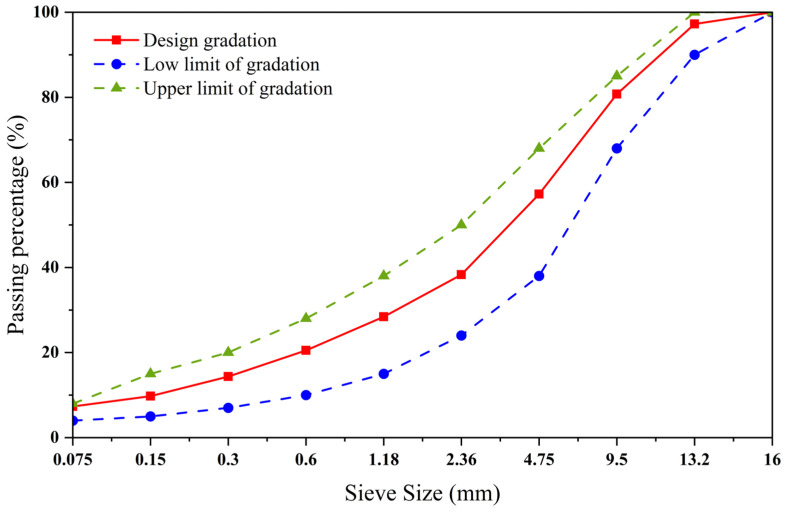
AC-type gradation curve graph.

The AC-13 type asphalt mixture uses limestone aggregates. The synthetic gradation curve of the aggregates used in the test is shown in Figure 5. Based on the results of the Marshall test presented in Table 7, the asphalt–aggregate ratio of the limestone AC-13 asphalt mixture was determined to be 5%. The mixing conditions for the asphalt mixture were set as 170 °C, 60 s, and 400 r/min [32].

(2) Preparation of SMA-13 Asphalt Mixture

**Figure 6:**
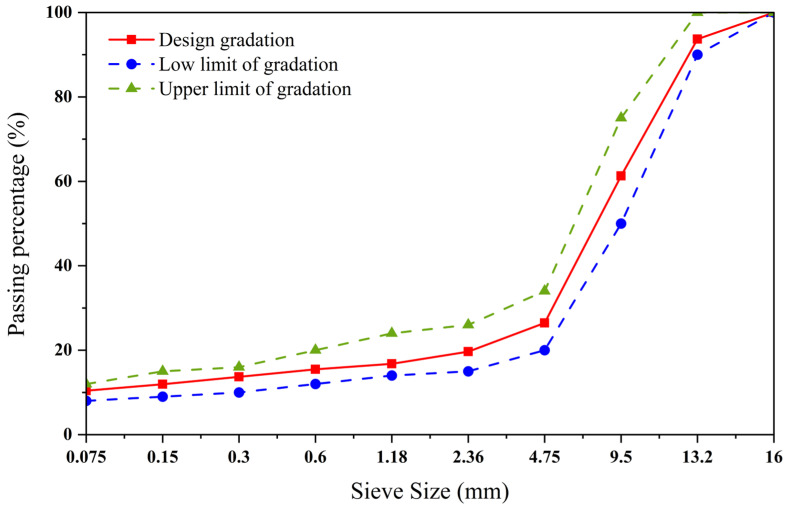
SMA-type gradation curve graph.

The SMA-13 type asphalt mixture uses basalt aggregates. The synthetic gradation curve of the aggregates used in the test is shown in Figure 6. The optimal asphalt–aggregate ratio of the asphalt mixture is known to be 6% based on THE literature [33]. The mixing conditions for the asphalt mixture are 165 °C, 60 s, and 300 r/min [34,35].

#### 2.1.5. Preparation of Microwave Asphalt Mixture

(1) Preparation of AC-type Microwave Asphalt Mixture

Following the equal-mass replacement method, AC-type microwave-modified asphalt mixtures (microwave-modified asphalt mixtures) were prepared by partially replacing mineral powder with magnetite powder, while maintaining the total mass of magnetite powder and mineral powder at 5% of the mineral aggregates. The dosages of magnetite powder were set to 0%, 0.1%, 0.2%, 0.5%, 1%, and 2% of the mineral powder mass, respectively, and the resulting microwave-modified asphalt mixtures were named wbAC-1, wbAC-2, wbAC-3, wbAC-4, wbAC-5, and wbAC-6, respectively.

(2) Preparation of SMA-type Microwave Asphalt Mixture

Following the equal-mass replacement method, SMA-type microwave-modified asphalt mixtures were prepared by partially replacing mineral powder with magnetite powder, while maintaining the total mass of magnetite powder and mineral powder at 10% of the mineral aggregates. The dosages of magnetite powder were set to 0%, 0.2%, 0.5%, 1%, and 2% of the mineral powder mass, respectively, and the resulting microwave-modified asphalt mixtures were named wbSMA-1, wbSMA-2, wbSMA-3, wbSMA-4, and wbSMA-5, respectively.

### 2.2. Performance-Testing Methods for Asphalt Mixtures

#### 2.2.1. Wave Absorption and Temperature Rise Test

The microwave absorption and heating test was conducted to evaluate the microwave absorption and heating performance of each microwave-modified asphalt mixture. A total of 300 g of the mixed microwave-modified asphalt mixture was placed in a laboratory microwave oven, and the temperature of the microwave-modified asphalt mixture was measured using an insert-type digital display thermometer, the supplier of this product is Hangzhou Shuce Technology Co., Ltd., with its city of location in Hangzhou and country of origin in China. Before microwave heating, the initial temperature of the microwave-modified asphalt mixture was measured. For the AC-type microwave-modified asphalt mixture, the temperature was measured every 2 min, while for the SMA-type microwave-modified asphalt mixture, the temperature was measured every 1 min. Heating was stopped when the temperature of the microwave-modified asphalt mixture rose to approximately 200 °C.

#### 2.2.2. High-Temperature Rutting Test

The rutting test was conducted to evaluate the high-temperature performance of the asphalt mixture. Referencing the Industry Standard of the People’s Republic of China JTG E20-2011 [36] Specifications for Asphalt and Asphalt Mixture Tests in Highway Engineering, a repeated load of 0.7 MPa was applied to the asphalt mixture specimen using a rutting test machine under dry conditions at 60 °C, with a wheel speed of 42 cycles per minute. During the test, the rut depth of the specimen was recorded, and the dynamic stability (DS) was calculated according to Formula (1) to evaluate the high-temperature rutting performance of the asphalt mixture.

(1) $$\mathrm{DS}=\frac{15\;\times \;N}{d_{60}\;-\;d_{45}}=\frac{42\;\times \;15}{d_{60}\;-d_{45}}$$

Among them, *DS* refers to dynamic stability with the unit of cycles per millimeter; *N* refers to the number of wheel load cycles per minute, which is 42 cycles per minute in this study; and *d*_45_ and *d*_60_ refer to the rut depths measured at 45 min and 60 min, respectively.

#### 2.2.3. Low-Temperature Bending Test

The low-temperature bending test was conducted to evaluate the low-temperature crack resistance of the asphalt mixture. With reference to the Industry Standard of the People’s Republic of China JTG E20-2011 [36] the test temperature was set to −10 °C, air bath cooling was adopted, the loading rate was 50 mm/min, the span length was 200 mm, and the mid-span loading method was used. The flexural strain was determined based on the maximum deflection at the mid-span and calculated using Formula (2):

(2) $$\varepsilon_{b}=\frac{6\;\times \;h\;\times \;d}{L^{2}}$$

Among them, *ε_b_* refers to the flexural tensile failure strain with the unit of με; h refers to the height of the specimen with the unit of mm; d refers to the vertical deflection of the specimen at the middle cross-section with the unit of mm; and L refers to the length of the support rollers with the unit of mm.

#### 2.2.4. Immersed Marshall Test

The Marshall stability test was conducted using a Marshall stability tester to evaluate the mechanical properties of the MMAM. The specimen was compressed at a speed of 50 mm/min until fracture. In accordance with the Industry Standard of the People’s Republic of China JTG E20-2011 [36] the specimen was immersed in water at 60 °C for 30 min before testing. For each MMAM, three specimens were prepared, respectively.

#### 2.2.5. Freeze–Thaw Cycle Splitting Tests and Beam Bending Tests

Multiple freeze–thaw cycle splitting tests (with the splitting test temperature set at 25 degrees Celsius) and multiple freeze–thaw cycle beam bending tests (with the bending test temperature set at minus 10 degrees Celsius) are used to evaluate the effect of the incorporation of wave-absorbing materials on the durability of asphalt mixtures.

## 3. Microwave Heating Simulation Study

### 3.1. Simulation Method and Model Construction

In this study, simulation research on microwave heating in the heat transfer physics field was conducted using the finite element software COMSOL Multiphysics. Two interfaces, namely “Electromagnetic Waves-Frequency Domain” and “Solid Heat Transfer”, were coupled, and relevant models were established for each AC-type microwave-modified asphalt mixture with reference to actual experimental conditions.

#### 3.1.1. Theoretical Model

The COMSOL simulation software is based on the theoretical basis of electromagnetism. It uses the finite element method to simulate the microwave-heating process of materials, and its electromagnetic field can be described using the Maxwell equations:

(3) $$\nabla \cdot E\;=\;\frac{\rho }{\varepsilon_{0}}$$

(4) ∇⋅B=0

(5) $$\nabla \;\times \;E=\;-\frac{\partial }{\partial t}B$$

(6) $$\nabla \;\times \;B=\mu_{0}J+\mu_{0}\varepsilon_{0}\frac{\partial }{\partial t}E$$

The frequency of microwave heating changes very rapidly. For the heat transfer process, short-term frequency changes have little impact on temperature. Therefore, the Fourier transform is further performed on the Maxwell equations to transform the time domain to the frequency domain, and its control over the heat transfer process is achieved. The Helmholtz equation is obtained through derivation:

(7) $$\nabla \;\times \;\mu_{r}^{-1}\left(\nabla \;\times \;E\right)\;-{\;k}_{0}^{2}\left(\varepsilon_{r}\;-\;j\frac{\sigma }{\omega E_{0}}\right)E\;=\;0$$

(8) $$k_{0}^{2}=\omega^{2}\mu_{0}\varepsilon_{0}$$

Among them, *k*_0_ refers to the wave number in vacuum; *E* refers to the electric field intensity; *B* refers to the magnetic field intensity; ε_0_ refers to the permittivity of free space; *ε_r_* refers to the relative permittivity; *μ_r_* refers to the relative permeability; *μ*_0_ refers to the permeability of free space; *t* refers to the heating time; *ω* refers to the angular frequency of microwaves; *σ* refers to the electrical conductivity; and *J* refers to the current density.

The power loss during the microwave heating process can be expressed in accordance with the Poynting theorem:

(9) $$Q=\frac{1}{2}\omega \varepsilon_{r}^{\prime \prime }\int_{V}|E{|}^{2}\mathrm{dV}$$

Among them, *Q* refers to the loss power; *εr*″ refers to the dielectric loss; *V* refers to the effective volume for microwave absorption.

The temperature value at each point of the asphalt mixture under microwave heating can be calculated using the heat conduction formula:

(10) $$\rho C_{p}u\cdot \nabla T\;=\;\nabla \cdot \left(k\nabla T\right)+Q$$

Among them, *ρ* refers to the density of the asphalt mixture; *C_p_* refers to the specific heat; *u* refers to the convection velocity; *k* refers to the thermal diffusivity; *T* refers to the temperature change.

#### 3.1.2. Simulation Model Construction

**Figure 7:**
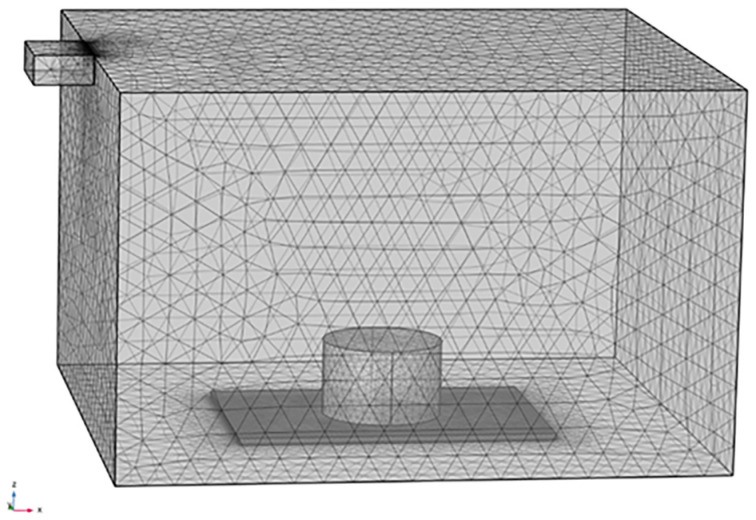
Mesh generation of microwave-heating system.

The microwave heating model refers to actual experimental conditions and consists of four parts: the microwave oven, the waveguide, the Marshall specimen of asphalt mixture, and the microwave-non-absorbing glass spacer. The microwave oven has a length of 510 mm, a width of 455 mm, and a height of 320 mm; the waveguide has a length of 50 mm, a width of 78 mm, a height of 25 mm, a microwave frequency of 2.45 GHz, and an input power of 700 W; the Marshall specimen of asphalt mixture has a diameter of 101.6 mm and a height of 63.5 mm; and the microwave-non-absorbing glass spacer has a length of 270 mm, a width of 180 mm, and a height of 6 mm. During the microwave heating process, the Marshall specimen is always placed at the center position of the glass spacer, and the glass spacer is placed at the middle position of the bottom of the microwave oven. To ensure result convergence and obtain relatively accurate calculation results, mesh generation is further performed on the model to create prismatic mesh elements, as shown in Figure 7.

### 3.2. Simulation of Microwave Heating of Asphalt Mixtures

This section aims to simulate the heating of microwave-modified asphalt mixtures in the microwave heating field using COMSOL software, specifically and intuitively study the heat conduction mechanism of magnetite powder in asphalt mixtures, and provide guidance for the implementation of actual experiments.

#### 3.2.1. Model Parameter Settings

In this model, the key parameters of magnetite are input into the COMSOL simulation in the following ways:

(1) The initial temperature of the geometric model is set to 20 °C (293.15 K); the microwave cavity is filled with air, and the physical parameters of the air are defined by the parameters in the built-in system material library of the COMSOL software; the boundary of the microwave cavity is treated as a perfect electric conductor; and the asphalt mixture specimen is treated as a homogeneous model.
(2) The electromagnetic parameters, such as permittivity and permeability, were derived and measured by means of the four-electrode method [37] based on the basic physical indicators of magnetite powder presented in Table 1. The thermal conductivity was determined in accordance with the (GB/T 5990-2006 [38]) [39]. Additionally, the heat capacity at constant pressure of the asphalt mixture specimen was tested using the method proposed by Grigoriev et al. [40].

The above steps ensure that the microwave absorption characteristics are consistent with those of actual materials. Among these parameters, the melting-point index meets the requirement that no phase change occurs during microwave heating. The specific parameters are shown in Table 8 and Table 9.

#### 3.2.2. Temperature Distribution of Microwave Asphalt Mixture

**Figure 8:**
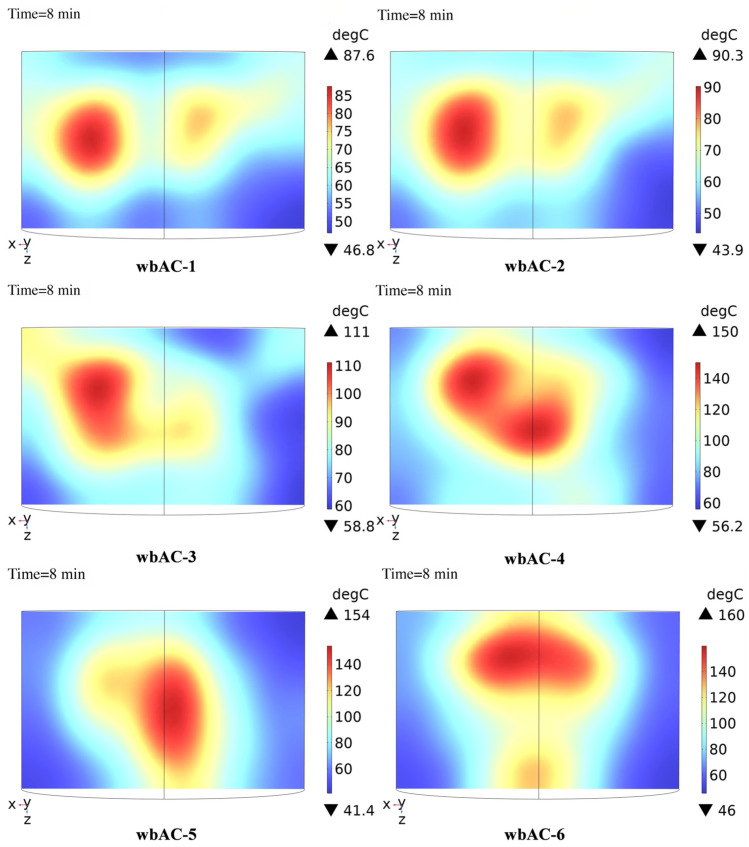
Numerically simulated temperature distribution of AC-type microwave asphalt mixture along the diametral section.

Temperature field simulation of microwave-modified asphalt mixtures is conducted using COMSOL software. After 8 min of heating, the typical internal temperature distribution diagrams of each microwave-modified asphalt mixture are shown in Figure 8.

As can be seen from Figure 8, the internal maximum temperature of the wbAC-4 microwave-modified asphalt mixture has significantly increased compared with that of the wbAC-1, wbAC-2, and wbAC-3 microwave-modified asphalt mixtures. While the increase in the internal maximum temperature of the wbAC-5 and wbAC-6 microwave-modified asphalt mixtures is relatively small compared with that of the wbAC-4 microwave-modified asphalt mixture, and there is no significant improvement in the overall temperature distribution of the wbAC-5 and wbAC-6 microwave-modified asphalt mixtures either.

**Figure 9:**
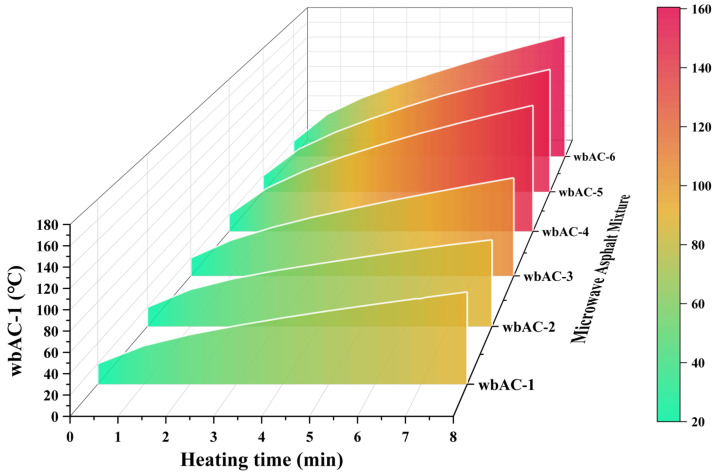
Temperature rise changes in microwave asphalt mixture.

The changes in internal temperature rise in each microwave asphalt mixture during the heating process within 0–8 min are shown in Figure 9.

As can be seen from Figure 9, within 0–8 min, the heating rates of the wbAC-1, wbAC-2, wbAC-3, wbAC-4, wbAC-5, and wbAC-6 microwave-modified asphalt mixtures are 8.45 °C/min, 8.7875 °C/min, 11.25 °C/min, 16.125 °C/min, 16.75 °C/min, and 17.5 °C/min, respectively. The heating effect of the wbAC-4 microwave-modified asphalt mixture is significantly improved compared with that of the wbAC-1, wbAC-2, and wbAC-3 microwave-modified asphalt mixtures, while the improvement in the heating effect of the wbAC-5 and wbAC-6 microwave-modified asphalt mixtures is relatively small compared with that of the wbAC-4 microwave-modified asphalt mixture.

## 4. Results and Discussion of Practical Experiments

### 4.1. Influence of Magnetite Powder on Microwave-Heating Effect of Asphalt Mixture

#### 4.1.1. Wave Absorption and Temperature Rise Performance of AC-Type Microwave Asphalt Mixture

**Figure 10:**
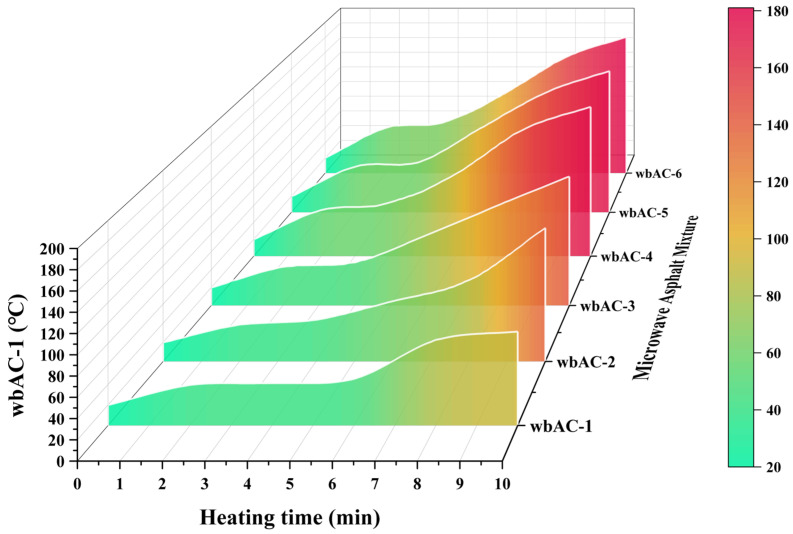
Temperature rise changes in AC-type microwave asphalt mixture.

The AC-type microwave-modified asphalt mixtures prepared in Section 2.1.5 are subjected to the microwave absorption and temperature rise test in accordance with the method described in Section 2.2.1. For each group of specimens, three parallel tests are conducted. The temperature change in the microwave-modified AC-13 asphalt mixture is shown in Figure 10.

As can be seen from Figure 10, the temperature of the AC-type microwave-modified asphalt mixtures increases with the increase in the content of magnetite powder. At the 8 min mark, which is consistent with the actual recycling temperature, the temperatures of the AC-type microwave-modified asphalt mixtures are 83.5 °C, 84.6 °C, 112.3 °C, 150.2 °C, 151.2 °C, and 154.3 °C, respectively. This indicates that adding magnetite powder can significantly enhance the microwave absorption and temperature rise performance of the AC-type microwave-modified asphalt mixtures. Specifically, when the content of magnetite powder is below 0.5%, the microwave absorption and temperature rise performance of the microwave-modified asphalt mixtures increases significantly with the increase in the content of magnetite powder; when the content of magnetite powder exceeds 0.5%, the magnitude of improvement in the microwave absorption and temperature rise performance of the microwave-modified asphalt mixtures decreases sharply. It can be concluded that 0.5% has reached the optimal content range of magnetite powder. Considering the cost factor comprehensively, the content of magnetite powder is finally determined to be 0.5% of the aggregate mass as the optimal scheme; that is, the wbAC-4 mixture is the optimal AC-type microwave-modified asphalt mixture.

#### 4.1.2. Microwave Absorption and Temperature Rise Performance of SMA-Type Microwave Asphalt Mixture

**Figure 11:**
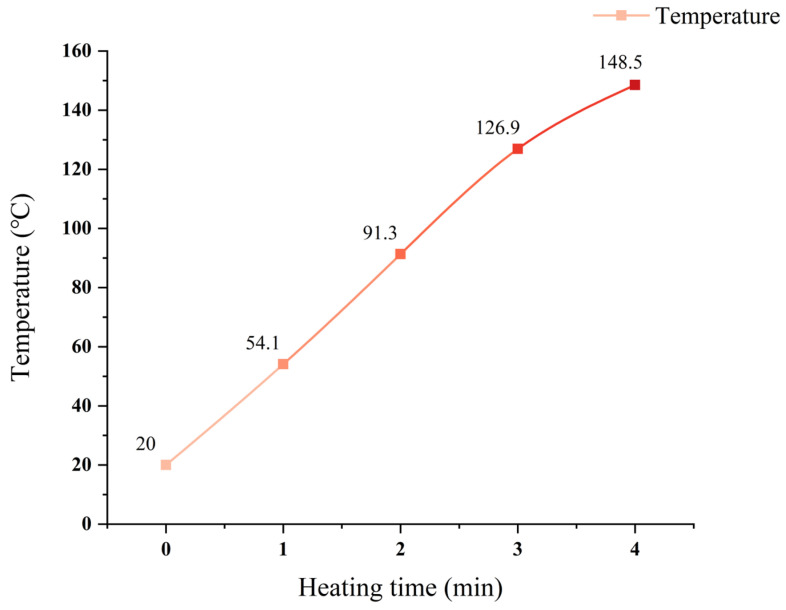
Temperature change in wbSMA-1 microwave asphalt mixture under 700 W power.

When the wbSMA-1 microwave-modified asphalt mixture is heated with a power of 700 W, the temperature change diagram of the wbSMA-1 microwave-modified asphalt mixture is obtained as shown in Figure 11. The temperature rise rate of the wbSMA-1 microwave-modified asphalt mixture is excessively high, and the data dispersion is relatively large. Therefore, the heating power of the microwave oven is adjusted to 420 W, and then the microwave absorption and temperature rise test is conducted on the SMA-type microwave-modified asphalt mixtures.

**Figure 12:**
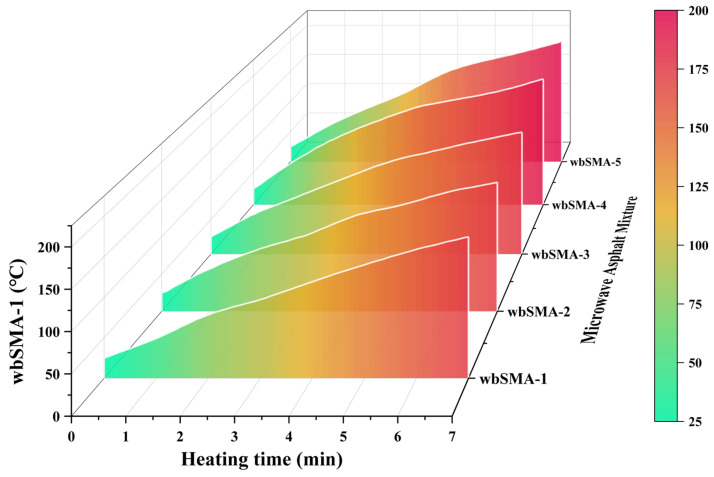
Temperature rise changes in SMA-type microwave asphalt mixture.

The SMA-type microwave-modified asphalt mixtures prepared in Section 2.1.5 are subjected to the microwave absorption and temperature rise test in accordance with the method described in Section 2.2.1. Three parallel tests are conducted for each group of specimens. The temperatures of the SMA-type microwave-modified asphalt mixtures at various time points are shown in Figure 12.

As seen in Figure 12, the temperature of the SMA-type microwave-modified asphalt mixtures increases with the increase in the content of magnetite powder. At the 5 min mark, which is consistent with the actual recycling temperature, the temperatures of the SMA-type microwave-modified asphalt mixtures are 140.1 °C, 142.5 °C, 148.7 °C, 164.1 °C, and 167 °C, respectively. This indicates that magnetite powder has a relatively small effect on improving the microwave absorption and temperature rise performance of the SMA-type microwave-modified asphalt mixtures. An analysis of the reasons shows that basalt aggregates themselves have relatively good microwave absorption performance; when the content of magnetite powder is low, the effect of improving the microwave absorption and temperature rise performance of the SMA-type microwave-modified asphalt mixtures is average. Specifically, when the content of magnetite powder is less than 0.5%, the magnitude of improvement in the microwave absorption and temperature rise performance of the SMA-type microwave-modified asphalt mixtures increases slowly with the increase in the content of magnetite powder; the microwave absorption and temperature rise performance of the SMA-type microwave-modified asphalt mixture with a magnetite powder content of 1.0% is optimized to a certain extent compared with that of the SMA-type microwave-modified asphalt mixture with a magnetite powder content of 0.5%; when the content of magnetite powder exceeds 1.0%, the upward trend of the microwave absorption and temperature rise performance of the SMA-type microwave-modified asphalt mixtures slows down again. Considering cost factors comprehensively, the content of magnetite powder is finally determined to be 1.0% as the optimal scheme; that is, the wbSMA-4 mixture is the optimal SMA-type microwave-modified asphalt mixture.

### 4.2. Explanation of the Mechanism of Magnetite Powder on the Microwave Heating Effect of Asphalt Mixtures

#### 4.2.1. Percolation Threshold Effect

Taking the AC-type microwave-modified asphalt mixture as an example, when the content of magnetite powder is lower than 0.5%, the magnetite particles are sparsely dispersed in the asphalt mixture and fail to form continuous electromagnetic loss paths, resulting in low microwave energy capture efficiency. At this point, an increase in magnetite powder content can significantly improve the connectivity of particles, enhance the integrity of loss paths, and greatly improve the microwave absorption and temperature rise performance. When the content reaches 0.5%, the distance between particles decreases to the critical value, a continuous loss network is formed, and the microwave energy absorption efficiency approaches the peak value. With a further increase in magnetite powder content, particle agglomeration destroys the uniformity of the loss network, leading to path breakage or local shielding, and the magnitude of improvement in microwave absorption performance decreases. Meanwhile, according to the data in Table 8, the dielectric loss ε″ of the wbAC-4 group with 0.5% magnetite powder content is 0.134, which is significantly higher than that of the group with 0.2% content (0.1054). However, the dielectric loss ε″ of the groups with 1% and 2% content decreases to 0.0981 and 0.0883, respectively. This change in data also proves that excessive magnetite particles will destroy the continuity of the absorption network, thereby leading to a reduction in absorption capacity.

#### 4.2.2. Magneto-Dielectric Loss Synergetic Saturation

Taking the AC-type microwave-modified asphalt mixture as an example, magnetite powder achieves synergistic microwave absorption through dielectric loss and magnetic loss. When the content of magnetite powder is lower than 0.5%, the density of loss sites is insufficient, resulting in a weak total loss effect; an increase in the magnetite powder content can linearly enhance the loss effect, and the temperature rise rate is significantly improved. When the content reaches 0.5%, the dielectric loss and magnetic loss reach the state of energy absorption saturation in the system, and the microwave-to-thermal energy conversion efficiency reaches the upper limit. At excessive content levels, excess particles cannot enhance the loss effect; instead, they disrupt the local electromagnetic field due to agglomeration, weakening the microwave absorption gain.

#### 4.2.3. The Impedance Matching Between Electromagnetic Parameters and Microwave Field

Taking the AC-type microwave-modified asphalt mixture as an example, the complex permittivity and tangent of loss angle of magnetite powder need to match the impedance of the asphalt mixture system to reduce microwave reflection. When the content of magnetite powder is 0.5%, the overall electromagnetic impedance of the mixture is in the transition region between air and the mixture, resulting in the minimum microwave reflection loss and the highest energy utilization efficiency. When the content of magnetite powder is lower than 0.5%, the microwave absorption capacity of the mixture is weak, and the microwave transmittance is high. When the content of magnetite powder exceeds 0.5%, agglomerated particles destroy the continuity of impedance, causing local microwave reflection and reducing the internal energy input.

Through the above theories, it can be effectively explained that a 0.5% magnetite powder content is the optimal content for the microwave absorption and temperature rise performance of AC-type microwave-modified asphalt mixtures. These theories are also applicable to SMA-type microwave-modified asphalt mixtures. Since the basalt aggregates used in SMA-type microwave-modified asphalt mixtures have good microwave absorption performance themselves, the magnetite powder particle density required for the mixture to form continuous electromagnetic loss paths, achieve magneto-dielectric loss synergetic saturation, and reach the optimal impedance matching of the air-asphalt mixture system is higher. Therefore, a 1% magnetite powder content is the optimal content for the microwave absorption and temperature rise performance of SMA-type microwave-modified asphalt mixtures.

### 4.3. Influence of Wave-Absorbing Materials on Pavement Performance of Asphalt Mixture

#### 4.3.1. High-Temperature Performance

**Figure 13:**
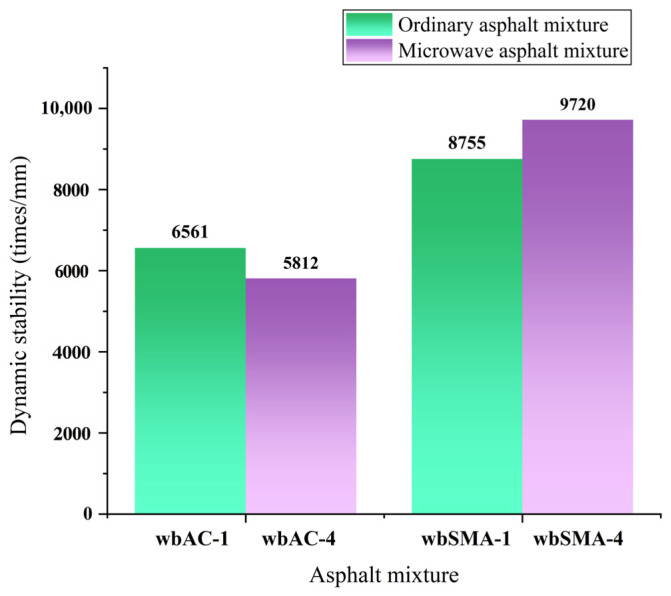
Results of high-temperature rutting tests on microwave-modified asphalt mixtures and ordinary asphalt mixtures.

By adopting the rutting test described in the previous sections, the effect of magnetite powder on the high-temperature performance of AC-type and SMA-type asphalt mixtures is studied. The results of the rutting test are shown in Table 10 and Figure 13.

As shown in Figure 13, after adding magnetite powder, the dynamic stability of the AC-type microwave-modified asphalt mixtures decreased by 11.4%, while the dynamic stability of the SMA-type microwave-modified asphalt mixtures increased by 5.88%. The Code for Design of Highway Asphalt Pavements (JTGD50-2017 [41]) requires that the dynamic stability of modified asphalt mixtures shall not be less than 3000, which proves that the high-temperature stability of the two types of microwave-modified asphalt mixtures prepared meets the requirements of the code.

#### 4.3.2. Low-Temperature Performance

**Figure 14:**
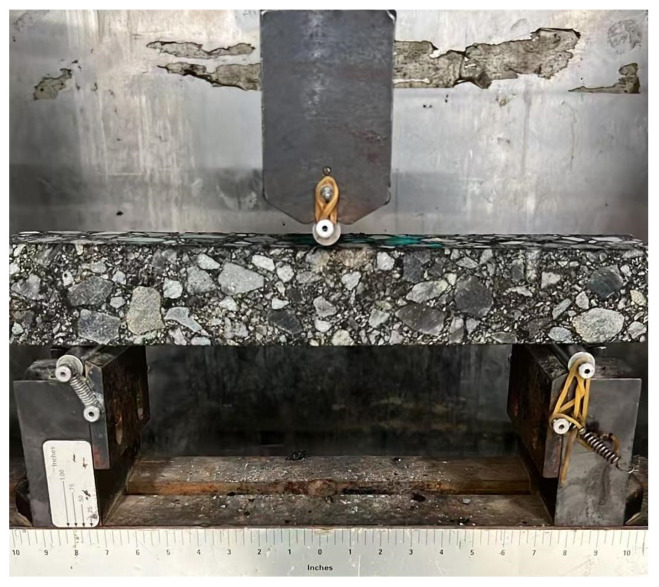
The beam specimen before the low-temperature test.

**Figure 15:**
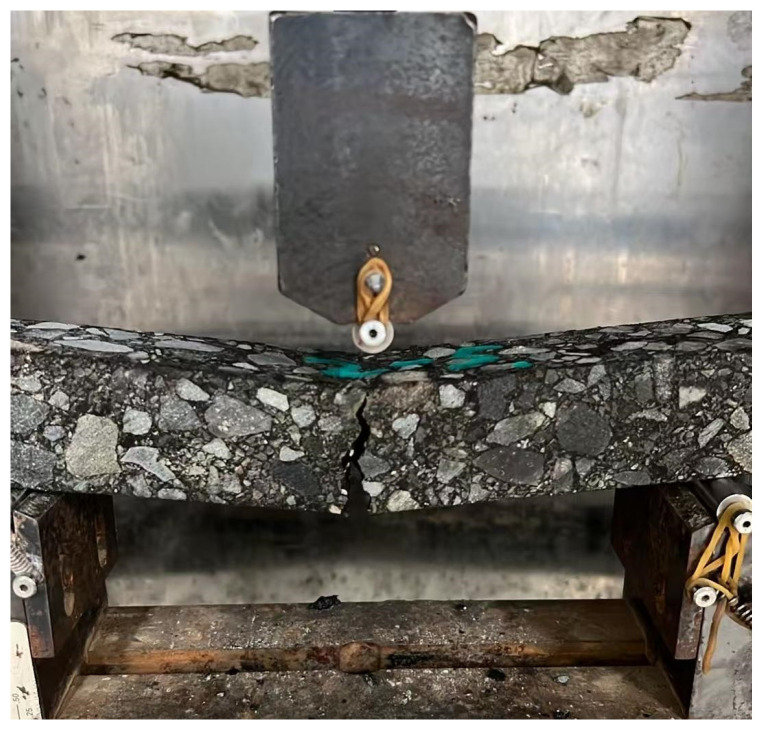
The beam specimen after the low-temperature test.

**Figure 16:**
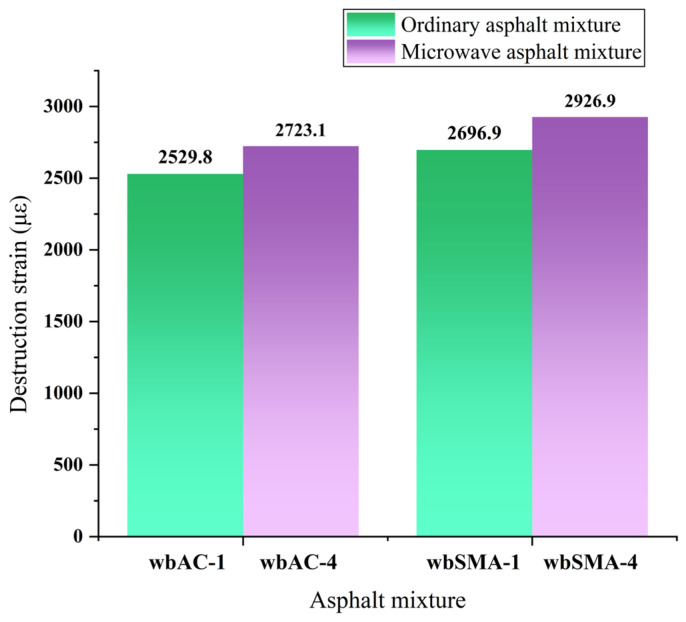
Results of low-temperature bending tests on microwave asphalt mixtures and traditional asphalt mixtures.

By adopting the low-temperature bending test described in the previous sections, the effect of adding magnetite powder on the low-temperature crack resistance of AC-type and SMA-type asphalt mixtures is studied. The beam specimens before and after the test are shown into Figure 14 and Figure 15. The results of the low-temperature bending test are shown in Table 11 and Figure 16.

As can be seen from Figure 16, after adding magnetite powder, the failure strain of the AC-type microwave-modified asphalt mixtures increased by 6.6%, and the failure strain of the SMA-type microwave-modified asphalt mixtures increased by 7.48%. Standard (JTGD50-2017 [41]) requires that the failure strain of modified asphalt mixtures shall not be less than 2500, which proves that adding magnetite powder has a positive effect on the low-temperature deformation resistance of asphalt mixtures and that the two types of microwave-modified asphalt mixtures can meet the requirements of the code.

#### 4.3.3. Water Stability Performance

**Figure 17:**
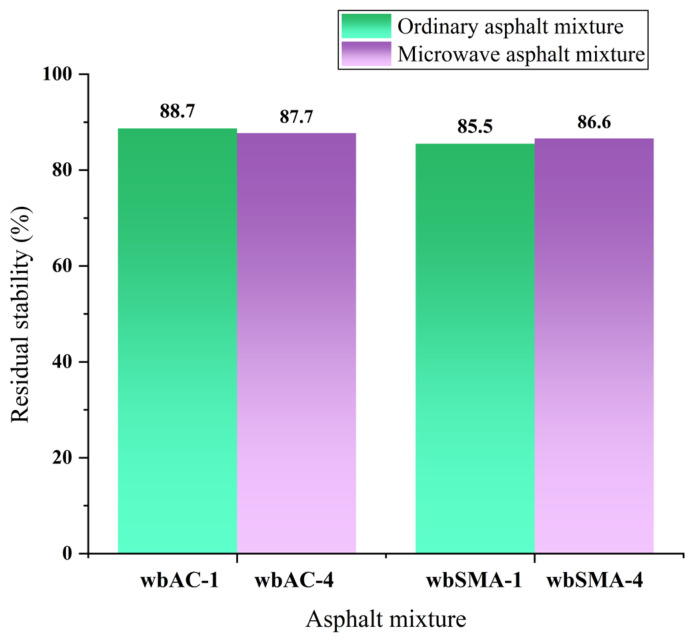
Results of immersed Marshall tests on microwave asphalt mixtures and traditional asphalt mixtures.

By adopting the immersed Marshall test described in the previous sections, the effect of magnetite powder on the water stability of AC-type and SMA-type asphalt mixtures is studied. The results of the immersed Marshall test are shown in Table 12 and Figure 17.

As can be seen from Figure 17, after adding magnetite powder, the water stability of the AC-type asphalt mixtures decreased by 1.12%, and the water stability of the SMA-type asphalt mixtures decreased by 2.14%. Standard (JTG D50-2017 [41]) specifies that the residual stability of modified asphalt mixtures shall not be less than 85. Therefore, the residual stability of the AC-type and SMA-type asphalt mixtures with added magnetite powder can meet the requirements of the code.

### 4.4. The Influence of Microwave-Absorbing Materials on the Durability Performance of Asphalt Mixtures

#### 4.4.1. Splitting Test After Freeze–Thaw Cycles

(1) Results and Analysis of Splitting Strength

**Figure 18:**
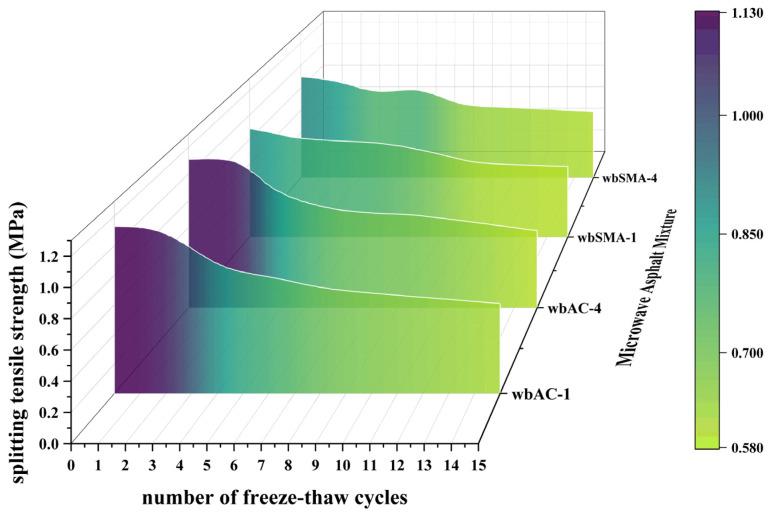
Freeze–thaw splitting tensile strength results.

This method uses cylindrical specimens compacted by the Marshall compaction method, with 50 compaction blows on each side, a diameter of 101.6 mm, and a height of 63.5 ± 1.3 mm. With reference to Standard (JTG E20-2011 [36]), the number of freeze–thaw cycles is set to 0 times, 2 times, 4 times, 6 times, 8 times, 10 times, and 15 times. The procedure of each cycle is as follows: place the specimens in a refrigerator at −18 °C for 16 h of freezing; take them out and immediately immerse them in a constant-temperature water bath at 60 °C for 24 h of thawing, thus completing one freeze–thaw cycle. After the completion of the cycles, cool the specimens to room temperature for subsequent use. Under an environment of 25 °C, splitting tests are conducted, with loading applied by a Marshall test apparatus at a loading rate of 50 mm/min. The maximum load at the time of specimen failure is measured, and the splitting tensile strength, freeze–thaw splitting strength ratio (TSR), and stiffness modulus are calculated. The test results are presented in Table 13 and Figure 18.

As can be seen from Table 13 and Figure 18, the freeze–thaw splitting tensile strength of asphalt mixtures gradually decreases with the increase in the number of freeze–thaw cycles and gradually tends to stabilize. The initial splitting strength of wbAC-1 is 1.13 MPa, that of wbAC-4 is 1.11 MPa, that of wbSMA-1 is 0.89 MPa, and that of wbSMA-4 is 0.90 MPa. Within six freeze–thaw cycles, the freeze–thaw splitting strength of AC-type asphalt mixtures is greater than that of SMA-type asphalt mixtures; when the number of freeze–thaw cycles exceeds six, the splitting tensile strengths of different types of asphalt mixtures are similar. From the comparative analysis of the freeze–thaw splitting strengths between ordinary asphalt mixtures and asphalt mixtures mixed with microwave-absorbing materials, it can be concluded that the addition of microwave-absorbing materials has little impact on the freeze–thaw cycle effect of asphalt mixtures.

**Figure 19:**
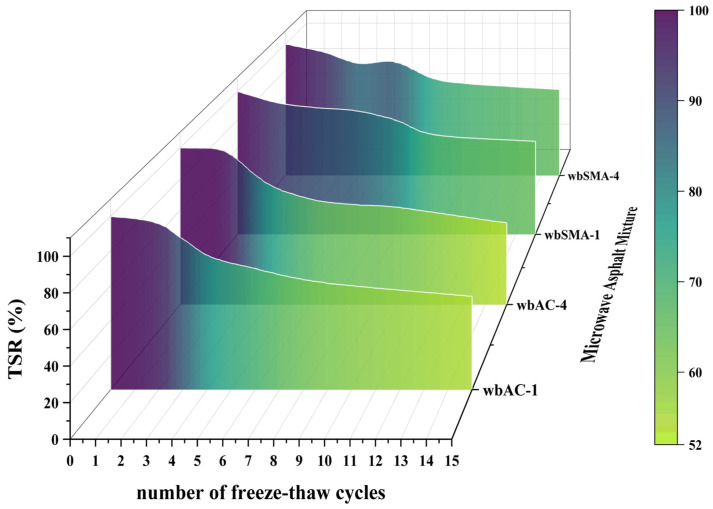
Freeze–thaw splitting tensile strength ratio results.

As can be seen from Table 14 and Figure 19, with the increase in the number of freeze–thaw cycles, the freeze–thaw splitting tensile strength ratio gradually decreases, and the freeze–thaw splitting tensile strength ratio of asphalt mixtures gradually tends to a constant value as the number of freeze–thaw cycles increases. As the number of freeze–thaw cycles for asphalt mixtures increases, the percentage of freeze–thaw splitting tensile strength (i.e., Freeze–Thaw Splitting Tensile Strength Ratio, TSR) shows little difference: after 4, 10, and 15 freeze–thaw cycles, the TSR values of wbAC-1 are 77.88%, 60.18%, and 53.98%, respectively; the TSR values of wbAC-4 are 77.48%, 62.16%, and 52.25%, respectively; the TSR values of wbSMA-1 are 88.76%, 69.66%, and 65.17%, respectively; and the TSR values of wbSMA-4 are 85.56%, 70%, and 65.56%, respectively. The TSR of SMA-type asphalt mixtures after freeze–thaw cycles is better than that of AC-type asphalt mixtures, and the addition of microwave-absorbing materials has little impact on the durability of asphalt mixtures.

(2) Analysis of Stiffness Modulus

The Poisson’s ratio of asphalt mixtures is taken as 0.40, and this value is used to calculate the fracture stiffness modulus of asphalt mixtures.

As can be seen from Table 15, the stiffness modulus gradually decreases with the increase in the number of freeze–thaw cycles and gradually tends to stabilize. When the numbers of freeze–thaw cycles are 4, 10, and 15, respectively, the stiffness modulus values of AC-type asphalt mixtures are 369.48 MPa, 331.8 MPa, and 315.23 MPa, respectively; the stiffness modulus values of SMA-type asphalt mixtures are 6450 MPa, 6350 MPa, and 6320 MPa, respectively.

To observe the rate of change in stiffness moduli of different types of asphalt mixtures after undergoing freeze–thaw cycles, and to calculate the percentage of initial stiffness modulus of asphalt mixtures after freeze–thaw cycles (i.e., the ratio of the stiffness modulus of the material after freeze–thaw cycles to the stiffness modulus of the material before freeze–thaw cycles), the results are presented in Table 16.

As can be seen from Table 16, the degree of decrease in the stiffness modulus of asphalt mixtures after freeze–thaw cycles is closely related to the types of asphalt mixtures, among which the degree of decrease for AC-type asphalt mixtures is relatively large, while that for SMA-type asphalt mixtures is relatively small. This indicates that SMA-type asphalt mixtures have better durability, and the addition of microwave-absorbing materials has a relatively small impact on the performance of asphalt mixtures.

#### 4.4.2. Low-Temperature Bending Test of Beam Specimens After Freeze–Thaw Cycles

**Figure 20:**
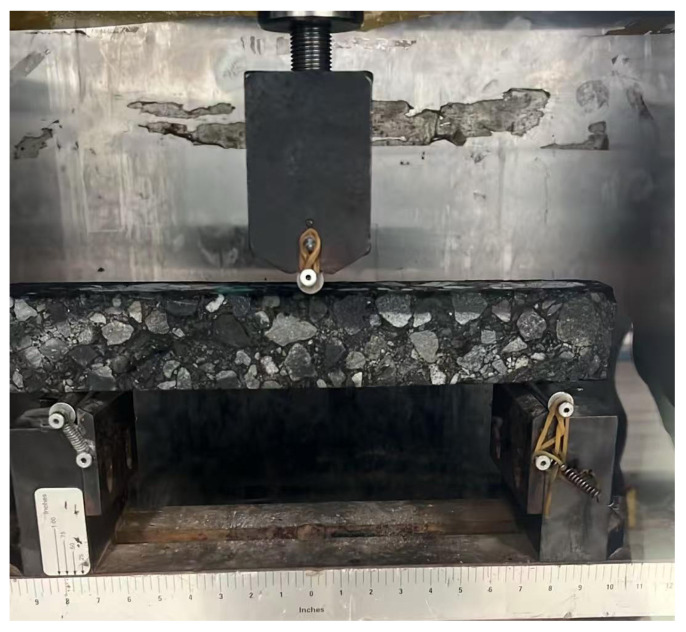
The beam specimen before the low-temperature bending test after freeze–thaw cycles.

**Figure 21:**
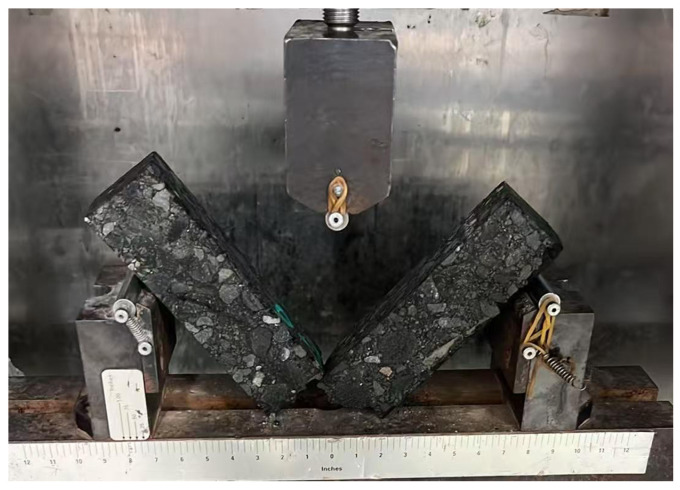
The beam specimen after thelow-temperature bending test after freeze–thaw cycles.

In the laboratory, low-temperature bending beam specimens are used to evaluate the performance of asphalt mixtures after different numbers of freeze–thaw cycles. Through tests, the maximum load at the time of low-temperature bending failure and the corresponding mid-span deflection of the beam specimens of different types of asphalt mixtures after different numbers of freeze–thaw cycles are measured. Furthermore, the flexural tensile strength, flexural tensile strain, and stiffness modulus of the beam specimens of different types of asphalt mixtures are obtained through calculations. The beam specimens before and after the test are shown in Figure 20 and Figure 21, and the calculation results are presented in Table 17.

As can be seen from Table 17, the flexural tensile strength and flexural tensile strain of both AC-type and SMA-type asphalt mixtures gradually decrease as the number of freeze–thaw cycles increases, with the flexural tensile strain showing a relatively larger degree of change with the number of freeze–thaw cycles. After 10 freeze–thaw cycles, the flexural tensile strain of the SMA-type asphalt mixture is 2406.2 με, and its flexural tensile strength is 9.82 MPa. Compared with the test group that did not undergo freeze–thaw cycles, which had a flexural tensile strain of 3418.5 με and a flexural tensile strength of 13.72 MPa, the decrease is relatively significant. The addition of microwave-absorbing materials has a relatively small impact on the asphalt mixture. Tests on the splitting strength and beam bending of different types of asphalt mixtures after freeze–thaw cycles indicate that the addition of an appropriate amount of microwave-absorbing materials does not induce a micro-expansion effect in the asphalt mixture.

### 4.5. Comprehensive Verification in Practical Environmen

#### 4.5.1. Test Preparation

**Figure 22:**
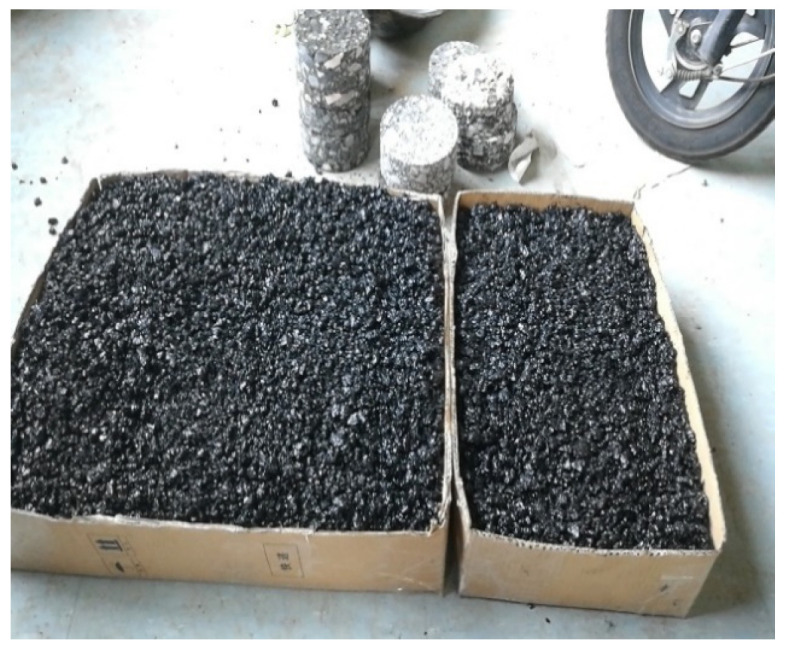
Formed asphalt mixture 1.

**Figure 23:**
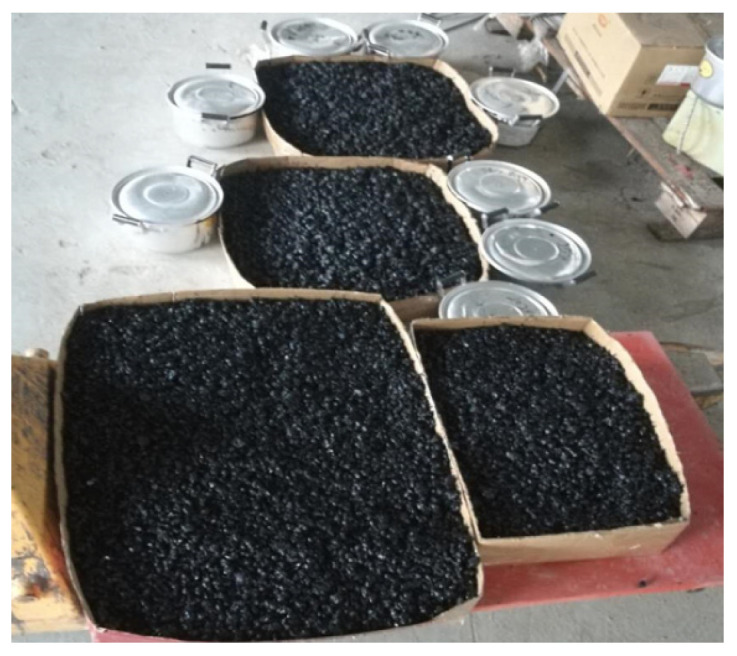
Formed asphalt mixture 2.

Based on the minimum heating area of 0.4 m^2^ (900 mm × 450 mm) of the daily microwave comprehensive maintenance vehicle, corresponding cartons are manually made, with dimensions of 900 mm × 450 mm × 115 mm. The uniformly mixed asphalt mixture is filled into the cartons, and this process is shown in Figure 22 and Figure 23. After the mixture cools down naturally, a heating test is conducted using the daily microwave comprehensive maintenance vehicle.

#### 4.5.2. Field Test

(1) Test Methods

Place the molded AC-13 asphalt mixture and SMA-13 asphalt mixture directly below the heating plate of the microwave comprehensive maintenance vehicle. The distance between the heating plate and the asphalt mixtures is set to 10 cm. An inserted digital display thermometer is used to collect the temperature changes at the 5 cm position inside the asphalt mixtures. Data collection for the AC-13 asphalt mixture is performed once every 5 min, and for the SMA-13 asphalt mixture once every 4 min. Later, due to the relatively fast temperature rise in the mixtures, the measurement interval is adjusted to once every 2 min after 8 min. Eight sets of data from different positions are obtained each time, and the average value is calculated.

**Figure 24:**
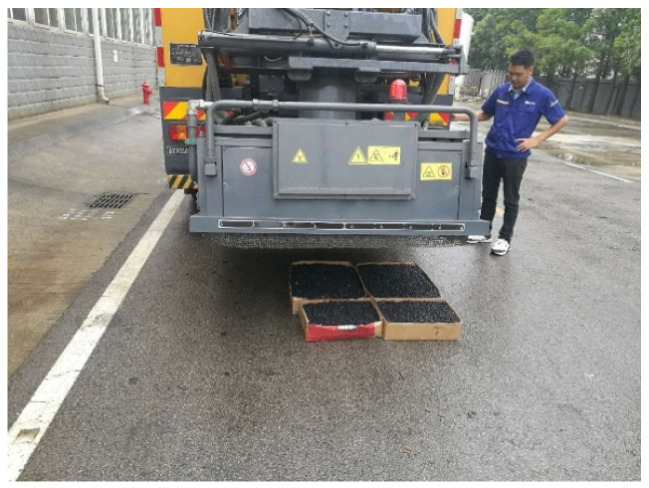
Microwave comprehensive maintenance vehicle.

**Figure 25:**
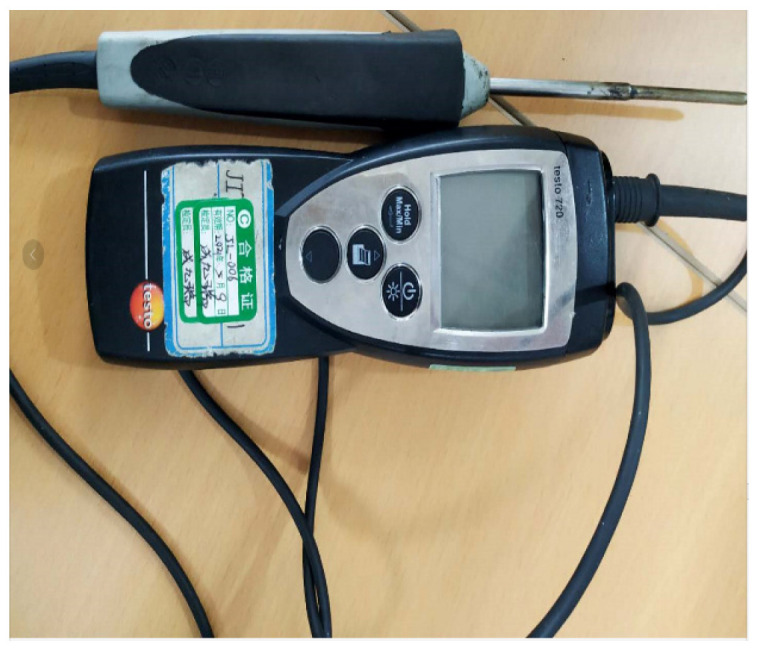
Inserted digital display thermometer.

**Figure 26:**
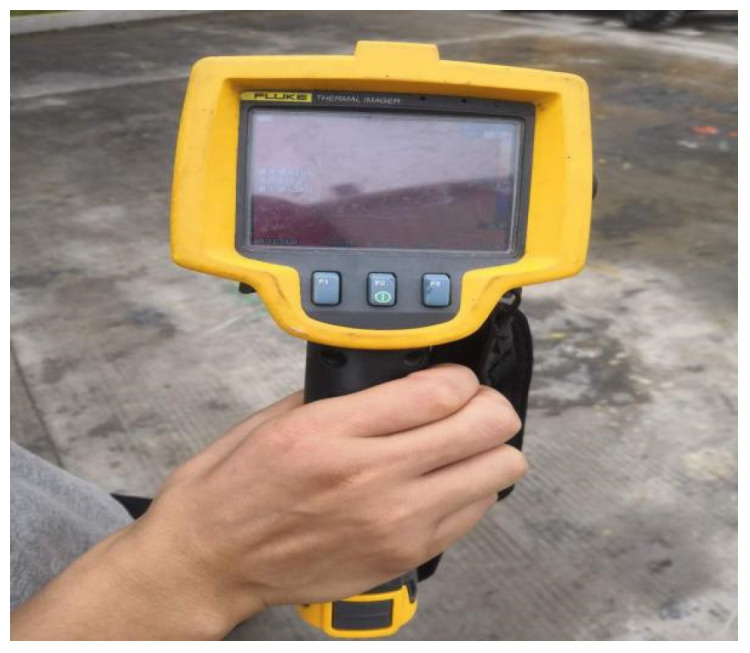
FLIR infrared thermal imager.

Second, the overall temperature test of asphalt mixtures during microwave heating uses infrared images of surface temperature obtained by an FLIR infrared thermal imager, and processes them using FLIR Tools software (FLIR Tools 6.x) to observe the temperature condition of the test specimens; the equipment and instruments are shown in Figure 24, Figure 25 and Figure 26.

(2) Test Results and Analysis

**Figure 27:**
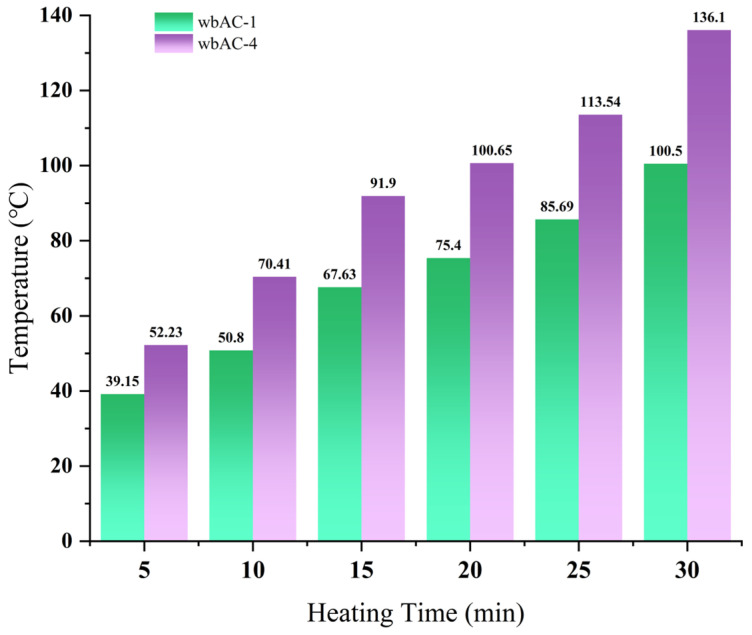
AC-13 mixture microwave-heating temperature change.

**Figure 28:**
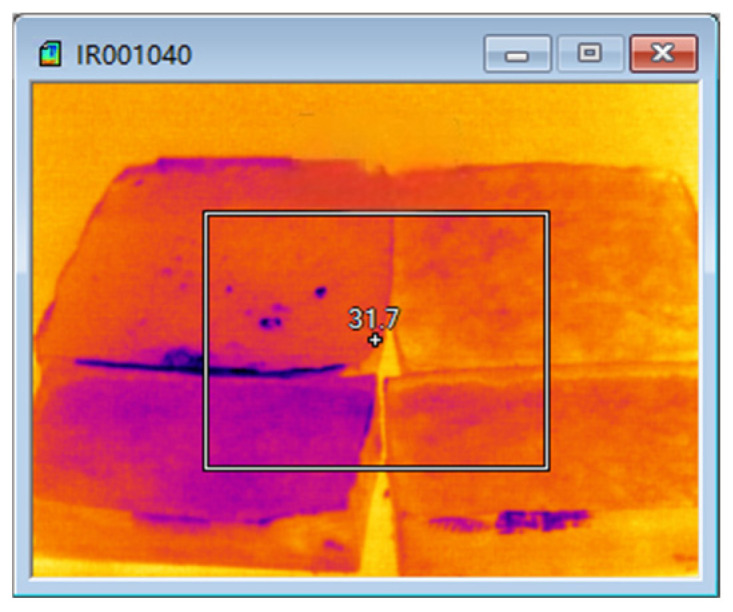
Before microwave heating.

**Figure 29:**
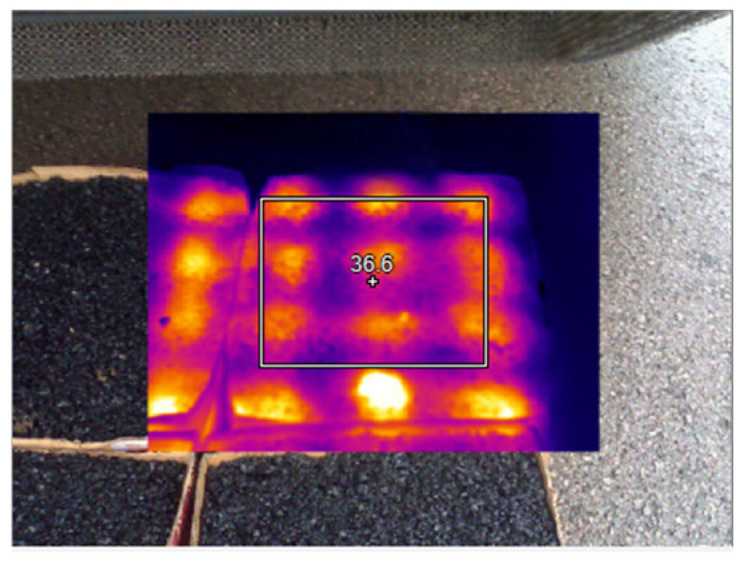
Microwave heating for 5 min.

**Figure 30:**
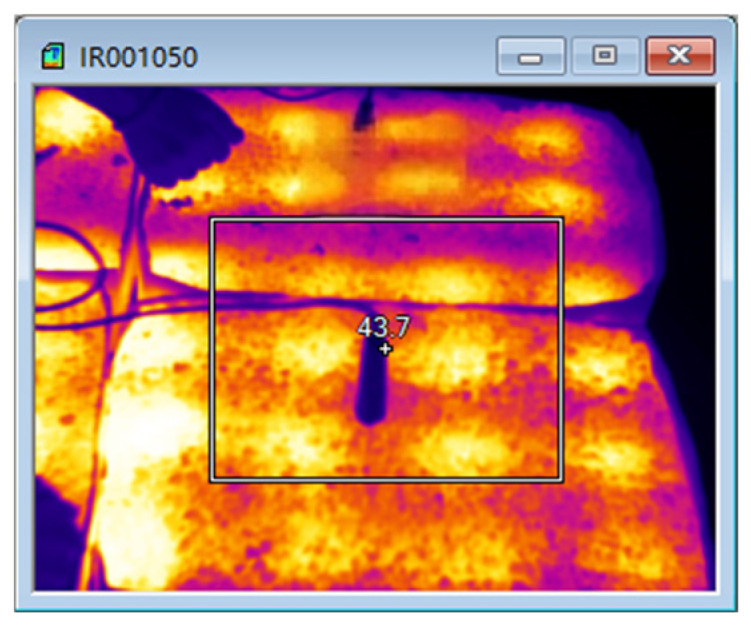
Microwave heating for 20 min.

**Figure 31:**
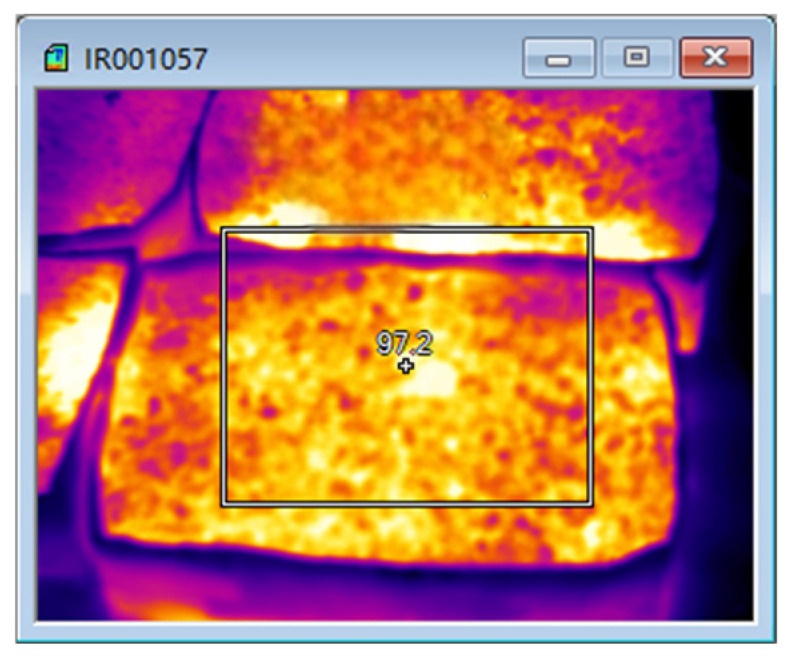
Microwave heating for 30 min.

The test results of microwave-heating temperatures for the AC-13 asphalt mixture at different heating times are shown in Table 18 and Figure 27, and the overall microwave-heating temperatures are shown in Figure 28, Figure 29, Figure 30 and Figure 31, with an initial temperature of 32 degrees Celsius.

The test results show that when the AC-13 asphalt mixture is heated by the microwave for 30 min, the temperature of the AC-13 asphalt mixture without magnetite powder is 100.5 °C, with a heating rate of 2.28 °C/min, while the temperature of the AC-13 asphalt mixture at the optimal content is 136.1 °C, with a heating rate of 3.47 °C/min, and the improvement in its heating rate reaches 52.2%.

**Figure 32:**
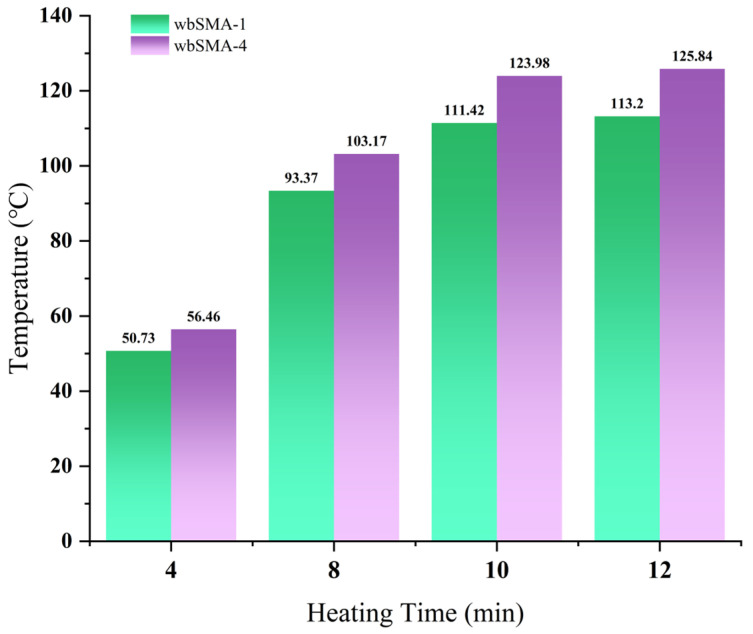
SMA-13 asphalt mixture microwave-heating temperature change.

**Figure 33:**
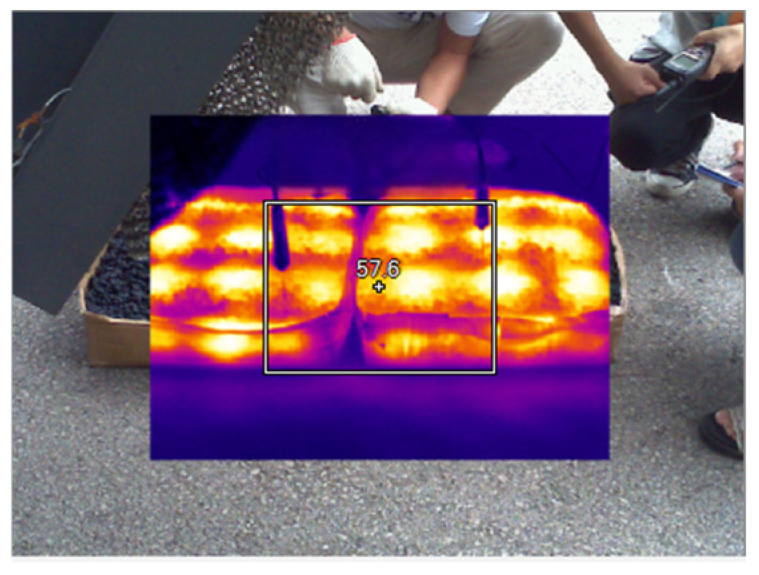
Microwave heating for 8 min.

**Figure 34:**
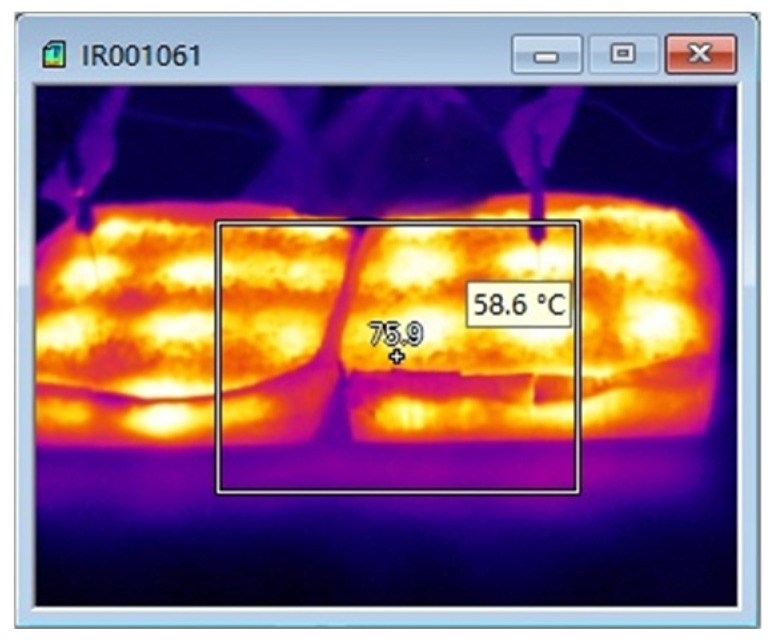
Microwave heating for 12 min.

The test results of microwave-heating temperatures for the SMA-13 asphalt mixture at different heating times are shown in Table 19 and Figure 32. The overall microwave-heating temperatures are shown in Figure 33 and Figure 34, with an initial temperature of 27.2 degrees Celsius.

The test results show that when the SMA-13 asphalt mixture is heated by the microwave for 12 min, the temperature of the SMA-13 asphalt mixture without magnetite powder is 113.24 degrees Celsius, with a heating rate of 7.17 degrees Celsius per minute, while the temperature of the SMA-13 asphalt mixture at the optimal content is 125.84 degrees Celsius, with a heating rate of 8.22 degrees Celsius per minute, and the improvement in its temperature reaches 14.6%.

From the comparison of temperature measurements between the infrared thermal imager and the inserted thermometer, it can be seen that the temperature values obtained from the point measurements of the inserted thermometer are slightly higher than those obtained from the infrared thermal imager. The main reason is that the surface temperature analyzed by the infrared thermal imager is the average temperature value of the entire surface of the asphalt mixture, while for the point measurement data, the temperature values of eight fixed points are selected, and their average value is used as the test data for analysis. Although the testing methods are different, both can reflect the temperature changes in the asphalt mixture caused by microwave heating.

## 5. Analysis of Sustainability and Carbon Emissions for Magnetite Powder Microwave Asphalt Mixtures

### 5.1. Sustainability Analysis

This study achieves sustainability in multiple dimensions for the application of asphalt mixtures. In the resource dimension, magnetite powder replaces ordinary mineral powder with equal mass; the optimal contents of magnetite powder in AC-type and SMA-type asphalt mixtures are 0.5% and 1% of the aggregate mass, respectively. This replacement does not require an additional increase in the total amount of aggregate, greatly reducing the demand for the extraction of primary aggregates. Moreover, magnetite powder is a natural mineral with abundant reserves, which is suitable for large-scale application. In the economic dimension, the low content of magnetite powder results in a negligible cost increase. After adding magnetite powder at the optimal contents, the failure strain of AC-type and SMA-type asphalt mixtures increases by 6.6% and 7.48%, respectively. Additionally, freeze–thaw tests show no significant decrease in durability, which can prolong the service life of pavements and reduce maintenance-related costs. In terms of environmental performance, magnetite powder has stable properties and no heavy metal pollution; it has a hydrophilic coefficient of 1.43, good compatibility with asphalt mixtures, and will not cause secondary pollution due to segregation.

### 5.2. Carbon Emissions Analysis

This study focuses on achieving carbon emission reduction in multiple aspects of asphalt mixtures: In the production stage, magnetite powder only requires physical crushing and does not need high-energy-consuming synthesis under high temperature and high pressure; in the heating-method stage, microwave heating uses electric energy as the source, produces no fuel combustion emissions, and can replace traditional infrared heating (which relies on fuel combustion to generate infrared radiation), thereby reducing carbon emissions during construction. In the heating-process stage, it can be concluded from the simulation and actual tests mentioned earlier that under the same heating power, the mixture mixed with magnetite powder has a faster temperature rise rate and consumes less electric energy when reaching the same temperature. Among them, at the optimal content, AC and SMA type asphalt mixtures reduce electric energy consumption by approximately 50% and 15%, respectively, thus achieving greater energy conservation.

## 6. Conclusions

This study takes natural magnetite powder as the wave-absorbing modifier, and explores its effects on the microwave-heating characteristics and pavement performance of AC-type and SMA-type asphalt mixtures through simulation, laboratory and field test evaluations, and sustainability analysis. It aims to provide a suitable scheme for on-site thermal recycling projects of pavement, and the core conclusions are as follows:

(1) Verified through COMSOL simulation and actual microwave heating tests, the optimal contents of magnetite powder in AC-type and SMA-type asphalt mixtures are 0.5% and 1% relative to the mass of the aggregate, respectively; at these contents, the wave absorption and temperature rise performances of the two types of mixtures increase by 52.2% and 14.6%, respectively, compared with those of ordinary asphalt mixtures.
(2) The key pavement performance indicators of the two types of mixtures under optimal contents are all in line with the requirements of the Specification for Design of Highway Asphalt Pavements (JTG D50-2017 [41]). The high-temperature dynamic stability of the AC-type microwave asphalt mixture decreases by 11.4%, while that of the SMA-type increases by 5.88%; the low-temperature failure strain of both increases by 6.6% and 7.48%, respectively, and their residual stability for water stability decreases by 1.12% and 2.14%, respectively. After 15 freeze–thaw cycles, their splitting tensile strength retention rate and stiffness modulus show extremely small differences from those of ordinary mixtures, and there is no durability degradation problem.
(3) Magnetite-powder-modified asphalt mixtures can achieve multi-dimensional sustainability in terms of resources, economy, and the environment, and their carbon emissions throughout the whole life cycle are significantly reduced. This not only meets the development needs of green transportation but also can provide strong support for the engineering promotion of in-place hot recycling technology.

## Figures and Tables

**Table 1:** Basic physical indices of magnetite powder.

Item	Test Results	Test Method/Tool
Fe_3_O_4_ content	95%	GB/T 6730.5-2016 [22] GB/T 6730.8-2016 [23]
Density (g/cm^3^)	4.5	GB/T 18711-2002 [24]
Particle size	2000 mesh	GB/T 19077.1-2016 [25]
Hydrophilic coefficient	1.43	JTG E42-2005 [26] T 0353 [27]
Melting point (°C)	1538	YS/T 1258-2018 [28]
Real part of complex permittivity/ε′	45.5	VNA
Imaginary part of complex permittivity/ε″	9.1	VNA
Loss tangent/tanδ = ε″/ε′	0.20	/

**Table 2:** Basic physical properties of dispersant and binder.

Item	Dispersant	Binder	Test Method
Name	Sodium hexametaphosphate (TAMOL-N)	Hydroxypropyl methylcellulose (HPMC)	/
Density (g/cm^3^)	2.484	1.39	GB/T 4472-2011 [29]
Particle size	500 mesh	200 mesh	GB/T 6003.1-1997 [30]
Melting point (°C)	616	/	GB/T 19466.3-2004 [31]

**Table 3:** Test results and technical indicators of SBS-modified asphalt.

Technical Parameters		Test Results	Technical Indicators
Penetration at 25 °C, 100 g, 5 s (0.1 mm)		66	50–80
Ductility at 5 cm/min, 5 °C (cm)		39	≥30
Softening Point (Ring and Ball Method) (°C)		71.5	≥60
Relative Density (g/cm^3^)		1.029	/
Flash Point (°C)		335	≥230
Residue after RTFOT (Rolling Thin Film Oven Test)	Mass Loss (%)	0.01	≤0.6
	Penetration Ratio (25 °C) (%)	71	≥65
	Ductility (5 cm/min, 5 °C) (cm)	31	≥20

**Table 4:** Test results and technical indicators of coarse aggregates.

Technical Parameters	(9.5–13.2) mm	(4.75–9.5) mm	Technical Indicators
Crushing Value (%)	18.5	/	≤20
Los Angeles Abrasion Loss Ratio (%)	11.3	12.4	≤28
Apparent Relative Density (g/cm^3^)	2.968	2.969	≥2.6
Needle-like Particle Content (%)	7.3	6.2	≤12
Water Absorption Rate (%)	0.66	0.79	≤2
Soundness (%)	4	3	≤12
Polished Stone Value (PSV) (BPN)	50	/	≥42

**Table 5:** Test results and technical indicators of fine aggregates.

Technical Parameters	Fine Aggregates	Technical Indicators
Apparent Relative Density (g/cm^3^)	2.972	≥2.5
Clay Content (Content of particles < 0.075 mm) (%)	2.3	≤3
Sand Equivalent (%)	66	≥60

**Table 6:** Test results and technical indicators of mineral powders.

Technical Parameters	Mineral Powder	Technical Indicators
Apparent Relative Density (g/cm^3^)	2.679	≥2.5
Specific Surface Area (m^2^/g)	1.389	/

**Table 7:** Results of Marshall test for designed gradation.

Type	Asphalt-Aggregate Ratio (%)	Bulk Relative Density of Specimen	Theoretical Maximum Relative Density	Void Ratio VV (%)	Voids in Mineral Aggregate VMA (%)	Voids Filled with Asphalt VFA (%)	Stability (kN)	Flow Value (0.1 mm)
AC-13	5.0	2.508	2.610	3.9	14.1	72.3	10.34	38.5
Requirements		-	-	3–5	-	65–75	≥8	20–40

**Table 8:** Electromagnetic parameters of simulation specimens.

Specimen Number	Real Part of Dielectric Constant	Imaginary Part of Dielectric Constant	Real Part of Permeability	Imaginary Part of Permeability
wbAC-1	6.41	0.082	1.0	0
wbAC-2	6.48	0.0945	1.0001	0
wbAC-3	6.67	0.1054	1.0001	0.001
wbAC-4	7.13	0.134	1.0002	0.0016
wbAC-5	7.61	0.0981	1.0003	0.0024
wbAC-6	8.02	0.0883	1.0005	0.0037

**Table 9:** Thermal parameters of simulation specimens.

Specimen Number	Density (kg/m^3^)	Heat Capacity at Constant Pressure (J/(kg⋅K))	Thermal Conductivity (W/(m⋅K))
wbAC-1	2712.3	945	1.346
wbAC-2	2713.4	922	1.325
wbAC-3	2714.5	898	1.307
wbAC-4	2717.6	855	1.211
wbAC-5	2722.9	827	1.203
wbAC-6	2733.7	803	1.198

**Table 10:** High-temperature rutting test data of microwave-modified asphalt mixtures and ordinary asphalt mixtures.

Types of Asphalt Mixture	Dynamic Stability (Passes/mm)					Coefficient of Variation (%)	
	1	2	3	Average	Requirements	Measured Values	Average
wbAC-1	6300	7326	6058	6561	≥2800	10.3	≤20
wbAC-4	5159	6630	5683	5812		9.5	
wbSMA-1	8750	9000	8514	8755	≥3000	2.8	≤20
wbSMA-4	9000	9265	9545	9270		2.9	

**Table 11:** Low-temperature bending test data of small beam specimens.

Types of Asphalt Mixture	Test Specimen Number	Maximum Load (kN)	Mid-Span Deflection (mm)	Flexural-Tensile Strength (MPa)	Stiffness Modulus (MPa)	Failure Strain (με)	Requirements (με)
wbAC-1	1	1.025	0.452	8.24	3280.0	2511.4	≥2500
	2	1.063	0.447	8.38	3312.7	2529.4	
	3	1.078	0.432	8.39	3344.5	2509.8	
	4	0.998	0.447	7.84	3048.5	2572.0	
	5	1.026	0.437	8.22	3238.4	2537.7	
	6	1.011	0.430	8.11	3218.3	2518.6	
	Average	1.034	0.441	8.20	3240.4	2529.8	
wbAC-4	1	1.060	0.515	8.52	3141.7	2711.5	
	2	1.067	0.510	8.46	3123.3	2708.1	
	3	1.114	0.504	8.67	3223.0	2691.4	
	4	1.076	0.516	8.45	3076.6	2747.7	
	5	1.082	0.500	8.55	3219.8	2655.0	
	6	1.080	0.501	8.43	3159.6	2667.8	
	Average	1.080	0.508	8.51	3157.3	2696.9	
wbSMA-1	1	1.360	0.509	10.97	4091.9	2679.9	≥2500
	2	1.409	0.532	11.37	4048.4	2809.0	
	3	1.378	0.519	11.30	4172.2	2709.2	
	4	1.372	0.497	11.24	4306.8	2609.3	
	5	1.422	0.511	11.60	4335.4	2675.1	
	6	1.396	0.544	11.40	3990.2	2856.0	
	Average	1.390	0.519	11.31	4157.5	2723.1	
wbSMA-4	1	1.253	0.502	9.79	3651.6	2973.7	
	2	1.139	0.506	9.43	3541.1	2957.1	
	3	1.331	0.498	10.75	4122.9	2900	
	4	1.284	0.482	10.04	3890.2	2874.1	
	5	1.197	0.497	9.01	3340.2	2991.7	
	6	1.280	0.483	10.40	4043.3	2865	
	Average	1.247	0.495	9.90	3764.9	2926.9	

**Table 12:** Immersed Marshall test data.

Types of Asphalt Mixture	Unconditioned (0.5 h)			Conditioned (48 h)			Residual Stability MS_0_ (%)	Requirements (%)
	Void Content (%)	Stability (kN)	Flow Value (0.1 mm)	Void Content (%)	Stability (kN)	Flow Value (0.1 mm)		
wbAC-1	4.3	10.62	35.4	4.2	9.17	42.7	88.7	≥85
	4.2	10.14	36.5	4.2	9.61	41.6		
	4.1	10.53	35.2	4.0	8.99	42.8		
Average	4.2	10.43	35.7	4.1	9.26	42.4		
wbAC-4	4.1	9.88	35.4	4.2	8.72	36.6	87.7	
	4.2	9.93	32.1	4.3	8.73	37.6		
	4.1	9.95	34.2	4.2	8.65	39.4		
Average	4.1	9.92	33.9	4.2	8.7	37.9		
wbSMA-1	4.0	11.23	36.2	4.1	10.17	35.7	85.5	≥85
	4.2	11.47	38.7	4.1	10.05	34.6		
	4.1	11.44	38.5	4.0	9.99	33.8		
Average	4.1	11.38	37.8	4.0	10.07	34.7		
wbSMA-4	3.8	7.10	34.3	3.7	6.34	40.2	86.6	
	3.7	7.45	34.2	3.8	6.02	41.3		
	3.7	7.92	35.0	3.6	7.11	41.0		
Average	3.7	7.49	34.5	3.7	6.49	40.8		

**Table 13:** Freeze–thaw splitting tensile strength data.

Number of Freeze–Thaw Cycles	wbAC-1 (MPa)	wbAC-4 (MPa)	wbSMA-1 (MPa)	wbSMA-4 (MPa)
0	1.13	1.11	0.89	0.90
2	1.08	1.09	0.82	0.85
4	0.88	0.86	0.79	0.77
6	0.79	0.75	0.77	0.78
8	0.72	0.71	0.71	0.67
10	0.68	0.69	0.62	0.63
15	0.61	0.58	0.58	0.59

**Table 14:** Freeze–thaw splitting tensile strength ratio data.

Number of Freeze–Thaw Cycles	wbAC-1 (MPa)	wbAC-4 (MPa)	wbSMA-1 (MPa)	wbSMA-4 (MPa)
0	100	100	100	100
2	95.58	98.20	92.13	94.44
4	77.88	77.48	88.76	85.56
6	69.91	67.57	86.52	86.67
8	63.72	63.96	79.78	74.44
10	60.18	62.16	69.66	70.00
15	53.98	52.25	65.17	65.56

**Table 15:** Splitting stiffness modulus of asphalt mixtures.

Number of Freeze–Thaw Cycles	wbAC-1 (MPa)	wbAC-4 (MPa)	wbSMA-1 (MPa)	wbSMA-4 (MPa)
0	390.85	387.75	6500	6450
2	380.56	376.85	6489	6400
4	369.48	365.45	6450	6387
6	356.78	351.75	6520	6350
8	342.23	340.78	6370	6340
10	331.8	332.53	6350	6300
15	315.23	310.51	6320	6278

**Table 16:** Stiffness modulus ratio after multiple freeze–thaw cycles.

Number of Freeze–Thaw Cycles	wbAC-1 (MPa)	wbAC-4 (MPa)	wbSMA-1 (MPa)	wbSMA-4 (MPa)
0	100	100	100	100
2	97.37	97.19	99.83	99.22
4	94.53	94.25	99.23	99.02
6	91.28	90.72	100.31	98.45
8	87.56	87.89	98.00	98.29
10	84.89	85.76	97.69	97.67
15	80.65	80.08	97.23	97.33

**Table 17:** Beam-bending-test data.

Type of Asphalt Mixtures	Number of Freeze–Thaw Cycles	Maximum Load (kN)	Mid-Span Deflection (mm)	Flexural Tensile Strength (MPa)	Stiffness Modulus (MPa)	Failure Strain (με)
wbAC-1	0	2.45	0.452	11.48	3177.9	3612.4
	5	2.31	0.447	10.82	2905.1	3724.5
	10	2.23	0.432	10.45	2737.6	3824.5
	15	2.29	0.447	10.73	2602.4	4123.1
wbAC-4	0	2.39	0.515	10.98	2887.6	3802.4
	5	2.26	0.510	10.56	2689.4	3926.5
	10	2.17	0.504	10.02	2529.2	3961.7
	15	2.18	0.516	10.15	2390.8	4245.5
wbSMA-1	0	2.93	0.509	13.72	4013.5	3418.5
	5	2.25	0.532	10.56	3967.5	2661.6
	10	2.09	0.519	9.82	4081.1	2406.2
	15	1.87	0.497	8.76	4306.8	2360.5
wbSMA-4	0	2.74	0.502	12.86	3651.6	2973.7
	5	2.12	0.506	9.95	3541.1	2957.1
	10	1.97	0.498	9.24	4122.9	2900
	15	1.85	0.482	8.69	3890.2	2874.1

**Table 18:** AC-13 asphalt mixture test results.

Material Type	5 min	10 min	15 min	20 min	25 min	30 min
wbAC-1	39.15	50.8	67.63	75.4	85.69	100.5
wbAC-4	52.23	70.41	91.9	100.65	113.54	136.1

**Table 19:** SMA-13 asphalt mixture test results.

Material Type	4 min	8 min	10 min	12 min
wbSMA-1	50.73	93.37	111.42	113.2
wbSMA-4	54.83	107.2	117.1	135.22

## Data Availability

The original contributions presented in this study are included in the article. Further inquiries can be directed to the corresponding author.
